# Prediction Intervals for Overdispersed Binomial Endpoints and Their Application to Toxicological Historical Control Data

**DOI:** 10.1002/pst.70033

**Published:** 2025-09-14

**Authors:** Max Menssen, Jonathan Rathjens

**Affiliations:** ^1^ Department of Biostatistics Leibniz University Hannover Hannover Germany; ^2^ Early Development Statistics Chrestos GmbH Essen Germany

**Keywords:** Bayesian hierarchical modeling, bootstrap‐calibration, long‐term carcinogenicity studies, micro‐nucleus‐test, OECD test guideline, Shewhart control chart

## Abstract

For toxicology studies, the validation of the concurrent control group by historical control data (HCD) has become requirements. This validation is usually done by historical control limits (HCL), which should cover the observations of the concurrent control with a predefined level of confidence. In many applications, HCL are applied to dichotomous data, for example, the number of rats with a tumor versus the number of rats without a tumor (carcinogenicity studies) or the number of cells with a micronucleus out of a total number of cells. Dichotomous HCD may be overdispersed and can be heavily right‐ (or left‐) skewed, which is usually not taken into account in the practical applications of HCL. To overcome this problem, four different prediction intervals (two frequentist, two Bayesian), that can be applied to such data, are proposed. Based on comprehensive Monte‐Carlo simulations, the coverage probabilities of the proposed prediction intervals were compared to heuristical HCL typically used in daily toxicological routine (historical range, limits of the np‐chart, mean ± 2 SD). Our simulations reveal, that frequentist bootstrap calibrated prediction intervals control the type‐1‐error best, but, also prediction intervals calculated based on Bayesian generalized linear mixed models appear to be practically applicable. Contrary, all heuristics fail to control the type‐1‐error. The application of HCL is demonstrated based on a real life data set containing historical controls from long‐term carcinogenicity studies run on behalf of the U.S. National Toxicology Program. The proposed frequentist prediction intervals are publicly available from the R package predint, whereas R code for the computation of the two Bayesian prediction intervals is provided via GitHub.

## 1 Introduction

### 1.1 Historical Control Data (HCD)

For several toxicological and pre‐clinical study types it is mandatory to store and report the outcome of the negative and sometimes also positive control groups of the experiments conducted before the current study (see e.g., OECD guidelines 471 [1], 487 [2], 489 [3] or EFSA [10]). In this context it is required to validate the concurrent control group by so called historical control limits (HCL) which, based on the HCD aim to predict the outcome of the current control group with x % (usually 95%) of confidence [4, 5, 6].

This approach is seen as a quality check for the current control group(s) and it is interpreted as a warning signal, if the current control is not covered by the HCL [7]: Either as a warning for a false‐positive result of the current study (if the concurrent control falls below the lower HCL) or as a warning for a false‐negative result (if the concurrent control falls above the upper HCL). With other words, it is desired that the HCL cover the central 95% of the possible values that can be obtained from the true underlying distribution.

Despite the fact, that the HCL based validation of a current control is required by several test guidelines (e.g., OECD 471 [1], OECD 473 [8], OECD 487 [2], OECD 490 [9]), most guidelines fail to provide reproducible methodology on how to compute HCL. Hence, many heuristics such as the historical range (the min and the max of the HCD) or the mean ± 2 standard deviations are used for the practical calculation of HCL, but suffer from severe theoretical drawbacks [6].

### 1.2 Calculation of HCL Based on Prediction Intervals

Contrary to the widely applied heuristics, prediction intervals directly aim to predict one (or more) value(s) from the same data generating process from which the historical data is derived. Hence several authors recommend the use of prediction intervals for the application of HCL [4, 5, 6, 10, 11, 12].

It has to be noted that the prediction intervals presented in several standard textbooks and other scientific publications [13, 14, 15, 16, 17] ground on the assumption that the historical sample is comprised of independent and identically distributed (iid) observations, meaning that possible between‐study variation is assumed to be absent. With other words, this simplistic prediction intervals do not account for the hierarchical design of the HCD, that is, they do not reflect that certain experimental units belong to a certain historical control group of a certain historical study.

Hence, the restriction to the iid assumption does not make sense in the context of toxicological HCD for which systematic between study variation is a fundamental part of the data generating process, and consequently, is regularly reported in real life data. For example, HC data sets that are prone to systematic between study variation can be found in [4, 18, 19, 20, 21, 22, 23] (for further details see section 4 of the Data S1).

### 1.3 Dichotomous Endpoints

In toxicology, several study types assess dichotomous endpoints: for example, carcinogenicity studies (rats with vs. rats without a tumor), the micro nucleus test (cells with vs. cells without a micronucleus) or the recently developed liquid microplate fluctuation Ames MPF test following [24] (numbers of wells with vs. number of wells without a color change).

It seems natural to model such data based on the binomial distribution. But, as described before, the hierarchical design of the HCD gives rise for systematic between‐study variation. Thus, the variability of the data will exceed the variance that can be modeled by the simple binomial distribution. This effect is called overdispersion (or extra‐binomial variation) and can either be modeled based on the quasi‐binomial assumption—which relies on the assumption that the between‐study overdispersion is constant—or based on the beta‐binomial distribution in which the between‐study overdispersion depends on the number of experimental units within each HC group (see Section 2).

In the following, we provide four different methods to compute prediction intervals for overdispersed binomial data: Two frequentist intervals that depend on normal approximation and bootstrap calibration as well as two Bayesian intervals of which one is based on Bayesian hierarchical modeling, whereas the other depends on the Bayesian version of a generalized linear random effects model.

The manuscript is organized as follows: The next section provides an overview about the modeling of overdispersed binomial endpoints. Section 3 provides an overview about the calculation of HCL. Several heuristical HCL that are used in daily toxicological routine are given in Section 3.1. The proposed prediction intervals are described in Sections 3.2 and 3.3. Section 4 reviews the properties of two real life HC data bases, that serve as an inspiration for Monte‐Carlo simulations regarding the coverage probabilities of the proposed methodology (Section 5). The application of the proposed methods is demonstrated in Section 6. The manuscript ends with a discussion (Section 7) and conclusions (Section 8).

## 2 Models for Overdispersed Binomial Data

One possible way to model overdispersion is the beta‐binomial distribution in which the binomial proportion for each of the h=1,…,H historical control groups is derived from a beta distribution

(1) $$\pi_{h}∼\text{Beta}\left(a,b\right)$$

and the random variable within each cluster is binomial

(2) $$Y_{h}∼\mathrm{Bin}\left(n_{h},\pi_{h}\right)$$

In this setting $E\left(\pi_{h}\right)=\pi =a/\left(a+b\right)$, $E\left(Y_{h}\right)=n_{h}\pi$. Furthermore, π is the overall binomial proportion and $n_{h}$ is the a priori known and fixed total number of experimental units per historical control group. The variance of the beta‐binomial distributed random variable is

(3) $$\mathrm{var}\left(Y_{h}\right)=n_{h}\pi \left(1-\pi \right)\left[1+\left(n_{h}-1\right)\rho \right]$$

In this parametrisation $\rho =1/\left(1+a+b\right)$ describes the intra‐class correlation coefficient [25]. Because the parameters of the beta distribution are restricted to be a>0 and b>0, also the intra‐class correlation is restricted to be 0<ρ<1.

Another possibility to model between‐study overdispersion is the quasi‐binomial approach in which the dispersion parameter ϕ constantly inflates the variance

(4) $$\mathrm{var}\left(Y_{h}\right)=n_{h}\pi \left(1-\pi \right)\phi$$

In the case in which the number of experimental unis within each control group becomes equal $n_{h}=n_{1}$, also the part of the beta‐binomial variance that controls the magnitude of overdispersion per control group $\left[1+\left(n_{h}-1\right)\rho \right]$ in Equation (3) becomes a constant. In this condition, the beta‐binomial model can be interpreted as a special case of the quasi‐binomial model—one in which the magnitude of the intra‐class correlation ρ together with the cluster size $n_{h}=n_{1}$ determines the magnitude of the dispersion parameter ϕ. Consequently, in this special case, both models coincide [11] and

(5) $$\phi \;\hat{=}\;\left[1+\left(n_{h}-1\right)\rho \right]\;\text{if}\;\mathrm{all}\;n_{h}=n_{1}$$

Both approaches are standard for modeling overdispersion [26], but in practice it might sometimes appear by chance, that a data set shows signs of underdispersion (less variability than possible under the simple binomial assumption). From Equation (3) it can be deduced that underdispersion is caused by negative correlations between the experimental units within each cluster (if the intra‐class correlation ρ was allowed to be negative). Practically this would mean that the chance to observe a further experimental unit with an event decreases, with the occurrence of an event (e.g., if one rat develops a tumor, the chance that the other rats in the cohort develop a tumor decreases). From a biological point of view, this is heavily implausible. Consequently the quasi‐binomial dispersion parameter was restricted to be ϕ≥1 in the following sections of this manuscript.

## 3 HCL for Overdispersed Binomial Data

The calculation of the HCL $\left[l,u\right]$ given below is based on the observed number of experimental units with an event $y_{h}$ out of a total number of experimental units per historical control group $n_{h}$ (e.g., rats with a tumor out of a total number of rats). All HCL are aimed to cover a further number of experimental units with an observed number of successes $y^{*}=y_{H+1}$ out of a further a priori known total number of experimental units $n^{*}=n_{H+1}$ with coverage probability

(6) $$P\left(l\leq y^{*}\leq u\right)=1-\alpha$$

Since HCL are computed to validate the current control group, the desired HCL should aim to cover the central $100\left(1-\alpha \right)\%$ of the underlying distribution and its limits should approach the lower α/2 and the upper 1−α/2 quantile, respectively (see fig. 1 of [27]). It is crucial to ensure for this behavior also, if the underlying distribution is skewed [28]. Consequently, the desired interval has to ensure for equal tail probabilities

(7) $$P\left(l\leq y^{*}\right)=P\left(y^{*}\leq u\right)=1-\alpha /2$$

This means that in practice, the control limits should cut off the lower and the upper $100\left(\alpha /2\right)\%$ of the underlying distribution, regardless if this distribution is symmetrical or skewed. For overdispersed binomial data, possible skewness depends on three factors:

1. The skewness increases, the closer π approaches its boundaries (0 or 1).
2. The skewness tends to increase with increasing between‐study overdispersion (depending on the value for π).
3. The skewness also tends to increases with decreasing cluster size $n^{*}$ (depending on the values for π and the amount of between‐study overdispersion).

This behavior is demonstrated in Figures S1 and S2 of the Supporting Information S1.

### 3.1 Heuristical HCL

Although several authors, dissuade from its use [5, 6, 10, 20, 29], HCL based on the historical range are frequently applied in daily toxicological routine [30, 31, 32]. Since such HCL are given by

(8) $$\left[l=\min\left(y_{h}\right),u=\max\left(y_{h}\right)\right]$$

they aim to cover *all* realizations from the underlying distribution and hence, do not aim for a proper approximation of its central $100\left(1-\alpha \right)\%$. Note that the min and max of the observed data provide very poor estimates for the true min and max of the underlying distribution, especially if the number of historical observations is low. This is because values in the center of the distribution have a higher chance to occur, whereas the occurrence of more extreme values in the tails of the distribution are less likely. Furthermore it has to be noted, that for binomial endpoints this interval is explicitly based on the assumption of constant cluster size $n_{h}=n^{*}\forall h=1,\ldots H$, because the interpretation of the number of observed events makes only sense in relation to the given cluster size (meaning that 5 out of 50 rats with a tumor have a fundamental different interpretation than 5 out of 100 rats). Hence, a sensible interpretation of the historical range is not possible, if the cluster size differs between the studies.

Another popular method for the calculation of control limits for dichotomous data are the limits used in Shewhart np‐charts

(9) $$\left[l,u\right]=n^{*}\bar{\pi }\pm k\sqrt{n^{*}\bar{\pi }\left(1-\bar{\pi }\right)}$$

with $\bar{\pi }=\frac{\sum_{h}y_{h}}{\sum_{h}n_{h}}$. In practical applications k is usually set to 2 or 3 in order to approximate the central 95.4% or 99.7% of the underlying distribution [33]. This HCL are strictly based on the assumption that the observations are independent realizations of the same binomial distribution which can be adequately approximated by a normal distribution with mean $n^{*}\bar{\pi }$ and variance $n^{*}\bar{\pi }\left(1-\bar{\pi }\right)$. Hence, the Shewhart np‐chart presumes that the law of large numbers is applicable, ignores the variability in $\bar{\pi }$, fails to account for overdispersion and fails to ensure for equal tail probabilities.

HCL that are based on the mean ± k standard deviations are given by

(10) $$\left[l,u\right]=\bar{y}\pm \mathrm{kSD}$$

with $\bar{y}=\frac{\sum_{h}y_{h}}{H}$ and $\mathrm{SD}=\sqrt{\frac{\sum_{h}{\left(\bar{y}-y_{h}\right)}^{2}}{H-1}}$. This type of HCL is based on a simple normal approximation that lacks an explicit assumption about the mean–variance relationship and hence heuristically allows for overdispersion [20]. Similar to HCL that are based on the historical range, the mean ± k standard deviations are explicitly based on the assumption of constant cluster size $n_{h}=n^{*}\;\forall \;h=1,\ldots ,H$, because for dichotomous endpoints, the number of observed experimental units with an event is only interpretable in the context of the total number of experimental units in the particular control group $n_{h}$. Hence, the application of HCL that are computed by the mean ± k standard deviations to dichotomous HCD with different cluster sizes should strictly be avoided (see section 2 of the Supporting Information S1).

### 3.2 Bootstrap Calibrated Prediction Intervals

Similarly to the prediction intervals for overdispersed count data of [20], the two frequentist prediction intervals below are based on the assumption that

$$\frac{n^{*}\hat{\pi }-Y^{*}}{\sqrt{\mathrm{var}\left(n^{*}\hat{\pi }\right)+\mathrm{var}\left(Y^{*}\right)}}\overset{\text{approx}.}{∼}N\left(0,1\right)$$

Here, $Y^{*}$ is the future random variable (of which $y^{*}$ is a realization), $\hat{\pi }$ is the binomial proportion estimated from the HCD and $n^{*}$ is the a priori known number of experimental units of the future control group and $n^{*}\hat{\pi }$ and $Y^{*}$ are assumed to be independent random variables.

Following Menssen and Schaarschmidt 2019 [11], a prediction interval which is based on the quasi‐binomial assumption is given by

(11) $$\left[l,u\right]=n^{*}\hat{\pi }\pm z_{1-\alpha /2}\sqrt{\frac{\hat{\phi }n^{*2}\hat{\pi }\left(1-\hat{\pi }\right)}{\sum_{h}n_{h}}+\hat{\phi }n^{*}\hat{\pi }\left(1-\hat{\pi }\right)}$$

with $\hat{\phi }>1$ as an estimate for the between‐study overdispersion.

If between‐study overdispersion is modeled based on the beta‐binomial distribution, the corresponding prediction interval is given by

(12) $$\left[l,u\right]=n^{*}\hat{\pi }\pm z_{1-\alpha /2}\sqrt{\left[\frac{n^{*2}\hat{\pi }\left(1-\hat{\pi }\right)}{\sum_{h}n_{h}}+\frac{\sum_{h}n_{h}-1}{\sum_{h}n_{h}}n^{*2}\hat{\pi }\left(1-\hat{\pi }\right)\hat{\rho }\right]+n^{*}\hat{\pi }\left(1-\hat{\pi }\right)\left[1+\left(n^{*}-1\right)\hat{\rho }\right]}$$

with $\hat{\rho }$ as an estimate for the intra‐class correlation coefficient. Details on methodology for the estimation of $\hat{\pi }$, $\hat{\phi }$ and $\hat{\rho }$ are given in Section 3.4.

As mentioned above, overdispersed binomial data can be heavily right‐ or left‐skewed, but the prediction intervals in Equations (11) and (12) are still symmetrical. Furthermore, they depend on normal approximation and hence, tend to have coverage probabilities below the nominal level, especially if the number of historical studies is low.

To overcome this problem, a bootstrap calibration algorithm (see box below), that individually calibrates both interval borders was applied. Note, that the idea behind this algorithm is similar to a Monte‐Carlo simulation regarding the prediction intervals coverage probability. Therefore, the bootstrap samples $y_{b}$ that mimic the HCD, as well the bootstrapped future observation $y_{b}^{*}$ are derived from the same data generating process (which depends on the estimates for the model‐parameters estimated from the HCD). The algorithm aims to provide substitutes $q_{l}$ and $q_{u}$ for the standard normal quantile used in Equation (11) and (12), such that possible skewness of the underling distribution is taken into account to ensure for equal tail probabilities and enhance the intervals coverage probability.

**Bootstrap Calibration of the Proposed Prediction Intervals**

1. Based on the historical data $y=\left(y_{1},\ldots ,y_{H}\right)$ find estimates for the model parameters $\hat{\pi }$ and $\hat{\phi }$ in the quasi‐binomial case and $\hat{\pi }$ and $\hat{\rho }$ in the beta‐binomial case
2. Based on these estimates, sample B parametric bootstrap samples $y_{b}$ following the same experimental design as the historical data (for sampling algorithms see section 3 of the Data S1)
3. Draw B further bootstrap samples $y_{b}^{*}$ that mimic the possible numbers of success in a future control group of size $n^{*}$ (using the same sampling algorithms as in step 2)
4. Based on $y_{b}$ obtain bootstrapped estimates for the model parameters (either ${\hat{\pi }}_{b}$ and ${\hat{\phi }}_{b}$ or ${\hat{\pi }}_{b}$ and ${\hat{\rho }}_{b}$) and calculate ${\hat{\mathrm{var}}}_{b}\left(n^{*}\hat{\pi }\right)$ and ${\hat{\mathrm{var}}}_{b}\left(Y^{*}\right)$
5. Calculate lower prediction borders $l_{b}=n^{*}{\hat{\pi }}_{b}-q_{l}\sqrt{{\hat{\mathrm{var}}}_{b}\left(n^{*}\hat{\pi }\right)+{\hat{\mathrm{var}}}_{b}\left(Y^{*}\right)}$, such that all $l_{b}$ depend on the *same* value for $q_{l}$.
6. Calculate the bootstrapped coverage probability ${\hat{\psi }}_{l}=\frac{\sum_{b}}{B}I_{b}\;\text{with}\;I_{b}=1\;\text{if}\;l_{b}\leq y_{b}^{*}\;\text{and}\;I_{b}=0\;\text{if}\;y_{b}^{*}<l_{b}$
7. Alternate $q_{l}$ until ${\hat{\psi }}_{l}\in \left(1-\frac{\alpha }{2}\right)\pm t$ with t as a predefined tolerance around $1-\frac{\alpha }{2}$
8. Repeat steps 5–7 for the upper prediction border with ${\hat{\psi }}_{u}=\frac{\sum_{b}}{B}I_{b}\text{ with }I_{b}=1\text{ if }y_{b}^{*}\leq u_{b}\text{ and }I_{b}=0\text{ if }u_{b}<y_{b}^{*}$
9. Use the corresponding coefficients $q_{l}^{\text{calib}}$ and $q_{u}^{\text{calib}}$ and the estimates obtained from the initial HCD (either $\hat{\pi }$ and $\hat{\phi }$ or $\hat{\pi }$ and $\hat{\rho }$) for interval calculation

$\begin{array}{l} {}[l=n^{*}\hat{\pi }-q_{l}^{\text{calib}}\sqrt{\hat{\mathrm{var}}\left(n^{*}\hat{\pi }\right)+\hat{\mathrm{var}}\left(Y^{*}\right)},\\ \;u=n^{*}\hat{\pi }+q_{u}^{\text{calib}}\sqrt{\hat{\mathrm{var}}\left(n^{*}\hat{\pi }\right)+\hat{\mathrm{var}}\left(Y^{*}\right)}] \end{array}$.

The bootstrap calibrated quasi‐binomial prediction interval is given by

(13) $$\begin{array}{c} \;[l=n^{*}\hat{\pi }-q_{l}^{\text{calib}}\sqrt{\frac{\hat{\phi }n^{*2}\hat{\pi }\left(1-\hat{\pi }\right)}{\sum_{h}n_{h}}+\hat{\phi }n^{*}\hat{\pi }\left(1-\hat{\pi }\right)}\\ u=n^{*}\hat{\pi }+q_{u}^{\text{calib}}\sqrt{\frac{\hat{\phi }n^{*2}\hat{\pi }\left(1-\hat{\pi }\right)}{\sum_{h}n_{h}}+\hat{\phi }n^{*}\hat{\pi }\left(1-\hat{\pi }\right)}] \end{array}$$

Similarly, the bootstrap calibration of the beta‐binomial prediction interval results in

(14) $$\begin{array}{c} \;[l=n^{*}\hat{\pi }-q_{l}^{\text{calib}}\sqrt{n^{*}\hat{\pi }\left(1-\hat{\pi }\right)\left[1+\left(n^{*}-1\right)\hat{\rho }\right]+\left[\frac{n^{*2}\hat{\pi }\left(1-\hat{\pi }\right)}{\sum_{h}n_{h}}+\frac{\sum_{h}n_{h}-1}{\sum_{h}n_{h}}n^{*2}\hat{\pi }\left(1-\hat{\pi }\right)\hat{\rho }\right]}\\ u=n^{*}\hat{\pi }+q_{u}^{\text{calib}}\sqrt{n^{*}\hat{\pi }\left(1-\hat{\pi }\right)\left[1+\left(n^{*}-1\right)\hat{\rho }\right]+\left[\frac{n^{*2}\hat{\pi }\left(1-\hat{\pi }\right)}{\sum_{h}n_{h}}+\frac{\sum_{h}n_{h}-1}{\sum_{h}n_{h}}n^{*2}\hat{\pi }\left(1-\hat{\pi }\right)\hat{\rho }\right]}] \end{array}$$

The applied bootstrap calibration is a modified version of the algorithm proposed by Menssen and Schaarschmdit 2022 [12] and has also been applied to calibrate prediction intervals for overdispersed count data [20]. The only difference to Menssen and Schaarschmdit 2022 [12] is, that both interval limits are calibrated individually, but the search for $q_{l}^{\text{calib}}$ and $q_{u}^{\text{calib}}$ depends on the same bisection procedure using a tolerance t=0.001.

### 3.3 Bayesian Modeling

Bayesian approaches provide an alternative estimation of prediction intervals [34]. Future observations as well as all model parameters are interpreted as random variables depending on each other. Thus, a posterior distribution of a future observation can be derived immediately within the parameter estimation process.

#### 3.3.1 Hierarchical Modeling

Bayesian hierarchical modeling depends on the beta‐binomial model that is given in Equations (1) and (2). In a Bayesian interpretation, the experiments' parameters $\pi_{h}$ are assumed to follow a Beta distribution, as a second level in a hierarchy [35].

A Beta prior in mean‐precision‐parametrization (Beta proportion distribution [36]) is applied:

$$\pi_{h}∼\text{Beta}\left(\mu ,\kappa \right)\;\forall \;h=1,\ldots ,H$$

with a non‐informative prior for the location hyperparameter $\mu \in \left(0,1\right)$. A weakly informative gamma prior $\kappa ∼\mathrm{Ga}\left(a,b\right)$ should be applied to represent a realistic domain of the precision hyperparameter κ>0 and, thus, to obtain a stable estimation.

The parameters' posterior distributions, given the data, that is, symbolically

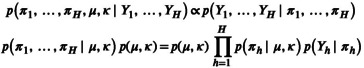

are estimated by Markov‐chain‐Monte‐Carlo (MCMC) sampling.

A posterior predictive distribution of a future observation $y^{*}$ is obtained by random sampling per MCMC iteration c=1,…,C: For every MCMC sample $\left({\tilde{\mu }}_{c},{\tilde{\kappa }}_{c}\right)$ of μ and κ, a prediction

$${\tilde{\pi }}_{c}^{*}∼\text{Beta}\left({\tilde{\mu }}_{c},{\tilde{\kappa }}_{c}\right)$$

of the future experiment's success proportion is drawn, and in turn a predicted future observation is sampled as

$${\tilde{y}}_{c}^{*}∼\mathrm{Bin}\left(n^{*},{\tilde{\pi }}_{c}^{*}\right)$$

A pointwise prediction interval for one future observation $y^{*}$ is then obtained using empirical quantiles of $\left\{{\tilde{y}}_{1}^{*},\ldots ,{\tilde{y}}_{C}^{*}\right\}$ such that

$$\left[l,u\right]=\left[q_{\alpha /2}\left(\left\{{\tilde{y}}_{1}^{*},\ldots ,{\tilde{y}}_{C}^{*}\right\}\right),q_{1-\alpha /2}\left(\left\{{\tilde{y}}_{1}^{*},\ldots ,{\tilde{y}}_{C}^{*}\right\}\right)\right]$$

in the two‐sided case.

#### 3.3.2 Bayesian Generalized Linear Mixed Model

A concept closely related to the Beta‐binomial model shown above is a generalized linear mixed model (GLMM), where Bernoulli distributed random variables $Z_{h1},\ldots ,Z_{{\mathrm{hn}}_{h}}$ with $\sum_{k=1}^{n_{h}}Z_{\mathrm{hk}}=Y_{h}$ are considered [37]. The success proportion $\pi_{h}=E\left(Z_{\mathrm{hk}}\right)$ is linked to a linear predictor

$$\eta_{h}=\ln\frac{\pi_{h}}{1-\pi_{h}}\in \left(-\infty ,+\infty \right)$$

which is comprised of a fixed general intercept ν and random effects $\beta_{h}$ per experiment:

$$\eta_{h}=\nu +\beta_{h},\;\beta_{h}∼N\left(0,\sigma \right)$$

With that, the historical experiments are again assumed to have individual success proportions $\pi_{h}$, derived from the same distribution, and the observations are again realizations of the binomial random variable $Y_{h}∼\mathrm{Bin}\left(n,\pi_{h}\right)$.

The posterior distributions of $\nu \in \left(-\infty ,+\infty \right)$ and σ>0 are estimated by MCMC sampling using non‐informative vague priors. Realizations of the future random variable $Y^{*}=\sum_{k=1}^{n^{*}}Z_{k}^{*}$ are derived: For every MCMC sample c, a prediction

$${\tilde{\beta }}_{c}^{*}∼N\left(0,{\tilde{\sigma }}_{c}\right)$$

of the future experiment's random intercept is drawn, followed by

$${\tilde{\eta }}_{c}^{*}={\tilde{\nu }}_{c}+{\tilde{\beta }}_{c}^{*}$$

Then, the future experiment's success proportion

$${\tilde{\pi }}_{c}^{*}=\frac{\exp\left({\tilde{\eta }}_{c}^{*}\right)}{\exp\left({\tilde{\eta }}_{c}^{*}\right)+1}$$

is derived, and in turn

$${\tilde{y}}_{c}^{*}∼\mathrm{Bin}\left(n^{*},{\tilde{\pi }}_{c}^{*}\right)$$

is drawn from the latter. Similarly as above, a pointwise two‐sided prediction interval for one future observation $y^{*}$ is given by

$$\left[l,u\right]=\left[q_{\alpha /2}\left(\left\{{\tilde{y}}_{1}^{*},\ldots ,{\tilde{y}}_{C}^{*}\right\}\right),q_{1-\alpha /2}\left(\left\{{\tilde{y}}_{1}^{*},\ldots ,{\tilde{y}}_{C}^{*}\right\}\right)\right]$$

### 3.4 Computational Details and Estimation

The proposed frequentist prediction intervals are implemented in the R package predint [6]. The uncalibrated prediction intervals (Equations (11) and (12)) are provided via qb_pi() and bb_pi(). The bootstrap calibrated prediction intervals (Equations (13) and (14)) are implemented via the functions quasi_bin_pi() and beta_bin_pi().

The estimates $\hat{\pi }$ and $\hat{\phi }$ used for the quasi‐binomial prediction interval were estimated based on a generalized linear model (stats::glm()). The estimates $\hat{\pi }$ and $\hat{\rho }$ for the beta‐binomial distribution were obtained following [25]. In order to avoid undersipersion, the quasi‐binomial interval was computed with $\hat{\phi }\geq 1.001$. The estimated intra‐class correlation used for the beta‐binomial interval was restricted to $\hat{\rho }\geq 0.00001$.

The bisection procedure used for bootstrap calibration is implemented in predint and provided via the function bisection(). This function takes three different lists as input, that contain the bootstrapped expected observations ${\hat{y}}_{b}^{*}$, the bootstrapped standard errors $\sqrt{\hat{\mathrm{var}}{\left(n^{*}\hat{\pi }\right)}_{b}+\hat{\mathrm{var}}{\left(Y^{*}\right)}_{b}}$ and the bootstrapped further observations $y_{b}^{*}$ and returns values for $q_{l}$ and $q_{u}$. Hence, this function enables the calculation of bootstrap calibrated prediction intervals in a general way, such that any Wald‐type prediction interval can be calibrated, as long as a parametric model can be fit to the data and a sampling function is available based on which parametric bootstrap samples can be generated.

The Bayesian hierarchical beta‐binomial model was applied using the software stan via the R package rstan [36, 38]. In the simulation, a weakly informative prior $\kappa ∼\mathrm{Ga}\left(2,5\cdot 10^{-5}\right)$ was used, giving the domain of the precision hyperparameter κ>0. The Bayesian GLMM was applied using the R function stan_glmer from the package rstanarm [39]. Functions for the computation of both Bayesian prediction intervals are provided via GitHub https://github.com/MaxMenssen/pi_overdisp_binomial/tree/main.

## 4 Properties of Real Life HCD

This section reviews the statistical properties of two real life HC data bases: One about long‐term carcinogenicity studies and one about the micronucleus test. The properties of the two data bases were taken into account for the setup of the Monte‐Carlo simulations regarding the coverage probability of the different HCL. This two study types were chosen in order to reflect the different properties of overdispersed binomial data at very different locations in the possible parameter space in order to enhance the simulations generalizability.

### 4.1 HCD From the Micro‐Nucleus Test

A micronucleus is a small fragment of chromosomes remaining outside the new nuclei after cell division. In the in vitro micro‐nucleus test (MNT), cell cultures on a well plate are treated with the compound of interest and an increased number of cells with a micronucleus is used as a proxy for genotoxicity [40]. Negative and positive control wells are observed to measure the spontaneous micronucleus proportion without treatment and to evaluate the test assay's functionality by comparison to HCD.

The analyzed real life HCD includes negative and positive control groups from about H≈50 experiments. Due to the high extend of standardization, the aggregated number of scored cells per control group is always $n_{h}=n^{*}=18000$.

The HC data base hints at an average proportion of cells with at least one micronucleus of about $\hat{\pi }\approx 0.01$ for negative control groups and about $\hat{\pi }\approx 0.15$ to 0.2 for positive controls. Overdispersion was estimated to range between $\hat{\phi }\approx 10$ and 50 for negative controls and tends to reach several hundreds for positive controls.

### 4.2 HCD From Long‐Term Carcinogenicity Studies

HCD on different endpoints obtained in long‐term carcinogenicity studies (LTC) are publicly available via the HC data base of the U.S. National Toxicology Program [41]. From this resource Menssen and Schaarschmidt 2019 [11] extracted HCD about the numbers of B6C3F1 mice per control group that had a hemangioma, the numbers of mice that showed at least one malignant tumor as well as the numbers of mice that passed away before the end of the study. This data was analyzed with regard to the binomial proportion $\hat{\pi }$ and the amount of overdispersion $\hat{\phi }$.

For rare events (hemangioma) the reported proportion was relatively low (close to zero) and overdispersion was estimated to be absent in most of the cases. The proportion of animals with malignant tumors ranged between 0.2 and 0.8, whereas the estimated dispersion parameters raised up to roughly four, meaning that the particular data set is four times as variable than possible under simple binomial distribution. The reported mortality rates behaved in a similar fashion. All reported control groups were comprised of 50 animals.

## 5 Simulation Study

In order to asses the coverage probabilities of the different methods for the calculation of HCL reviewed above, Monte‐Carlo simulations were run. In all simulations, the nominal coverage probability was set to 1−α=0.95. The parameter combinations used for the simulation were inspired by the real life data shown above. For all simulations the cluster size was fixed ($n_{h}=n^{*}\;\forall \;h=1,\ldots ,H$) such that the variance–mean relationships of the beta‐binomial distribution and the quasi‐binomial assumption coincide.

For each combination of model parameters, S=5000 “historical” data sets were drawn, based on which HCL ${\left[l,u\right]}_{s}$ were calculated. Furthermore, S sets of single “future” observations $y_{s}^{*}$ were sampled and the coverage probability of the control intervals was computed to be

(15) $${\hat{\psi }}^{\mathrm{cp}}=\frac{\sum_{s=1}^{S}I_{s}}{S}\;\text{with}$$

$$\begin{array}{c} I_{s}=1\;\text{if}\;y_{s}^{*}\in {\left[l,u\right]}_{s},\\ I_{s}=0\;\text{if}\;y_{s}^{*}\notin {\left[l,u\right]}_{s} \end{array}$$

Average interval borders were calculated as the mean of the simulated interval borders $\bar{l}=\frac{\sum_{s=1}^{S}l_{s}}{S}$ and $\bar{u}=\frac{\sum_{s=1}^{S}u_{s}}{S}$. Each single bootstrap calibrated prediction interval that was calculated in the simulation was based on B=10000 bootstrap samples. Each Bayesian prediction interval was calculated based on C=5000 MCMC samples.

### 5.1 Coverage Probabilities for the MNT‐inspired Simulation Setting

This part of the simulation was inspired by the properties of the real live HCD for the micro‐nucleus test reviewed above. 72 combinations of values for the number of historical studies H, the binomial proportion π and the amount of overdispersion ϕ were run, of which 18 combinations directly reflect the properties of the real life HCD (combinations of the bold faced parameter values in Table 1), whereas the remaining 54 combinations of parameter values were run in order to enhance the generalisability of the simulation.

**TABLE 1:** Parameter values used for the MNT‐inspired simulation.

Parameter	Values^a^
No. of historical studies (H)	**5**, **10**, **20**, 100
Binomial proportion (π)	0.001, **0.01**, **0.1**
Overdispersion (ϕ)^b^	1.001, 3, 5, **10**, **50**, **500**
Historical cluster size ($n_{h}$)	**18,000**
Future cluster size ($n^{*}$)	**18,000**

*Note:* Bold numbers: Parameter values that reflect properties of real life HCD.

^a^ Simulations were run for all 72 combinations of the given parameter values.

^b^ Since the cluster sizes $n_{h}$ and $n^{*}$ are constant $\phi \;\hat{=}\;1+\left(n_{h}-1\right)\rho \;\hat{=}\;1+\left(n^{*}-1\right)\rho$. The given values of ϕ correspond to intra‐class correlation coefficients ρ of 5.5e‐08, 0.00011, 0.00022, 0.00050, 0.00272, 0.02772.

Note that, because the cluster size was relatively huge ($n_{h}=n^{*}=18000$) most parameter combinations result in a relatively symmetrical underlying distribution (except for small proportions π=0.001 combined with high overdispersion ϕ=500, see Figure 1 in Supporting Information S1).

**FIGURE 1:**
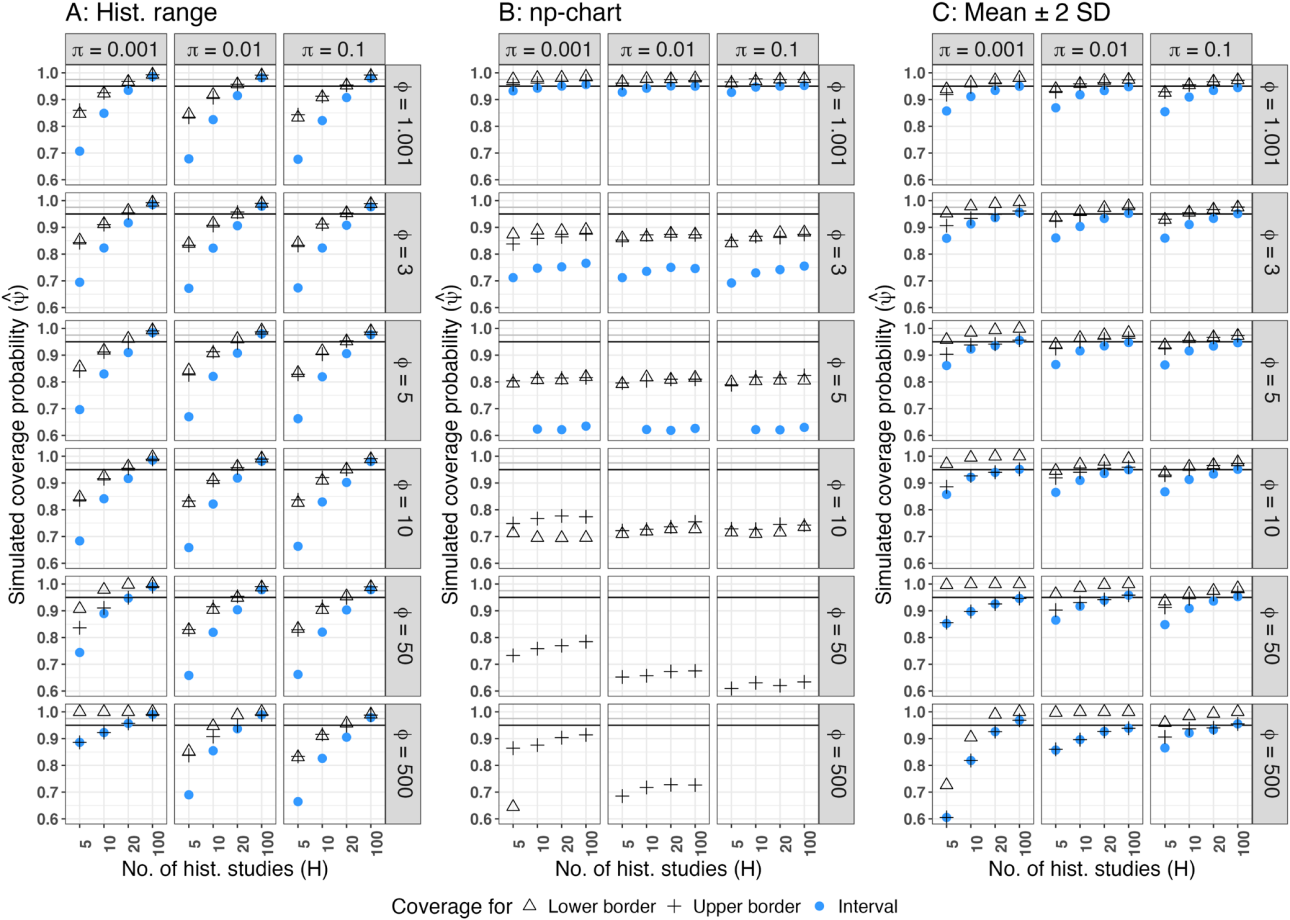
Simulated coverage probabilities of heuristical methods (MNT‐setting). (A) Historical range, (B) Np‐chart, (C) Mean ± 2 SD, *Black horizontal line*: Nominal coverage probability $\psi^{\mathrm{cp}}=0.95$, *Grey horizontal line*: Nominal coverage probability for the lower and the upper limit, if equal tail probabilities are achieved $\psi^{l}=\psi^{u}=0.975$. Coverage probabilities below 0.6 are excluded from the graphic.

**FIGURE 2:**
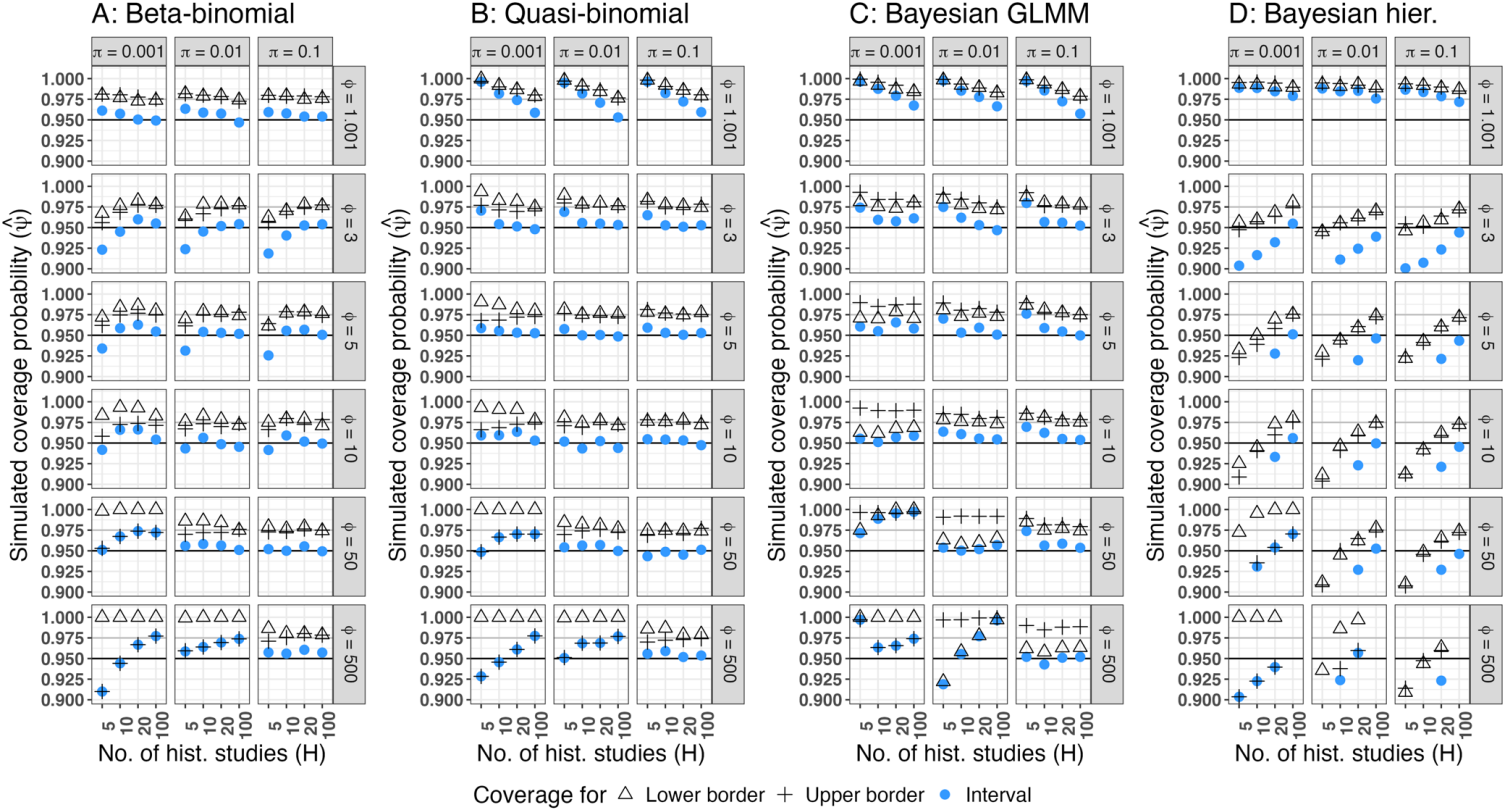
Simulated coverage probabilities of prediction intervals (MNT‐setting). (A) Calibrated beta‐binomial prediction interval, (B) Calibrated quasi‐binomial prediction interval, (C) Prediction interval obtained from a Bayesian generalized linear mixed model, (D) Prediction interval obtained from a Bayesian hierarchical model, *Black horizontal line*: Nominal coverage probability $\psi^{\mathrm{cp}}=0.95$, *Grey horizontal line*: Nominal coverage probability for the lower and the upper limit, if equal tail probabilities are achieved $\psi^{l}=\psi^{u}=0.975$. Coverage probabilities below 0.9 are not shown. The Bayesian hierarchical model faced serious convergence problems for ϕ=500 and H=100. Hence, coverage probabilities could not be computed in this setting.

**FIGURE 3:**
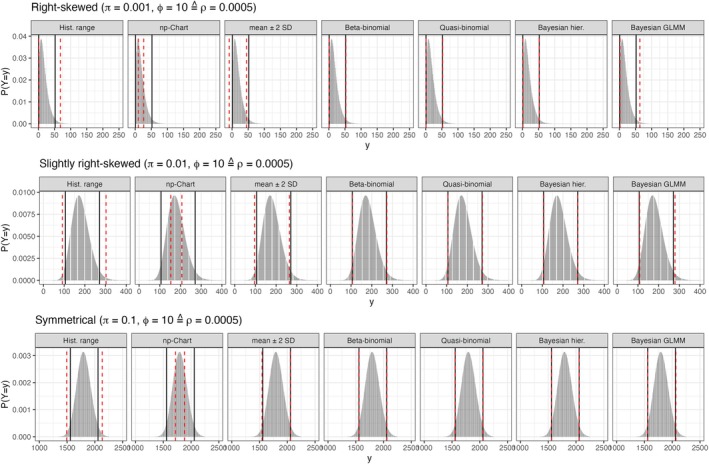
Average limits versus true quantiles of the underlying distribution (MNT‐setting). *Grey area*: Probability mass function of the underlying beta binomial distribution. *Black lines*: True underlying 2.5% and 97.5% quantiles. *Dashed red lines*: Average limits for H=100 and $n_{h}=18000$ obtained from the simulation.

The simulated coverage probabilities of the heuristical methods are depicted in Figure 1, whereas the coverage probabilities of the proposed prediction intervals are presented in Figure 2. Furthermore, simulated average interval borders and the corresponding true quantiles are given in Figure 3 for three simulation settings, that reflect different levels of possible skewness of the underlying distribution. In these simulation settings H was set to 100 historical control groups. Thus, the historical information is high enough, that obvious flaws of the methods should be clearly visible. None of the heuristics control the central 95% of the underlying distribution, whereas all prediction intervals properly converge to the true quantiles, regardless if the underlying distribution is skewed or not.

As expected, the historical range (Figure 1A) covers the simulated future observations $y_{s}^{*}$ in all the cases, if the amount of historical control groups is high enough and lacks a proper control of the statistical error (here 5%). Therefore, the application of the historical range for the calculation of HCL should be avoided.

If overdispersion is practically absent (ϕ=1.001), the control limits of the np‐chart (Figure 1B) yield coverage probabilities relatively close to the nominal level. But, with increasing overdispersion, their coverage probabilities decrease below 0.2, meaning that, by far, less than the desired central 95% of the underlying distribution are covered. This is also demonstrated in Figure 3: The average limits of the np‐chart are covered by the true quantiles. Consequently, the np‐chart cannot be recommended for the application to toxicological (overdispersed) HCD.

At first glance, it seems that the HCL computed by the mean ± 2 SD (Figure 1C) yield coverage probabilities close to the nominal level of 0.95, if the amount of historical control groups is high enough (at least 20). But, with a decreasing binomial proportion π and/or an increasing amount of overdispersion ϕ the right‐skewness of the data increases. Hence, in many cases the lower border almost always covers the future observation (the triangles in Figure 1C approach 1) whereas the coverage probability of the upper border (crosses) does not approach the desired 0.975. This behavior demonstrates that HCL computed based on the mean ± 2 SD do not ensure for equal tail probabilities, or in other words: They do not control the central 95% of the underlying distribution, if this distribution is skewed. This behavior is also demonstrated in Figure 3: The simulated average limits are systematically below the corresponding true quantiles, if the underlying distribution is right‐skewed.

The coverage probabilities of the prediction intervals proposed in Sections 3.2 and 3.3 are depicted in Figure 2. In general, both frequentist prediction intervals and the interval based on a Bayesian GLMM yield coverage probabilities relatively close to the nominal level for most settings. All three intervals behave in a comparable fashion, except for settings in which the number of historical control groups is low and/or the amount of overdispersion is high (H<10, ϕ=500). Contrary to this, the prediction interval drawn from a Bayesian hierarchical model, fails to control the statistical error in many of the cases. Furthermore, Bayesian hierarchical models faced serious convergence issues, if the amount of historical studies and the amount of overdispersion was high (H=100, ϕ=500), meaning that the desired interval could not be derived for many of the simulated data sets. Consequently, the coverage probability could not be computed in this three cases (which explains the missing dots for H=100 and ϕ=500 in Figure 2D).

In the (practical) absence of overdispersion (ϕ=1.01), the beta‐binomial prediction interval controls the alpha error best, whereas the prediction intervals based on the quasi‐binomial assumption or Bayesian GLMM tend to behave conservatively, but approach the nominal coverage probability with an increase of historical control groups (H=100). Contrary to this, the prediction interval obtained from Bayesian hierarchical modeling remains conservative in this setting, even for 100 historical studies.

For low to moderate proportions $\pi \in \left[0.001,0.1\right]$ and low to moderate overdispersion $\phi \in \left[3,50\right]$ (moderate in the context of $n_{h}=n^{*}=18000$), all intervals, except the one based on Bayesian hierarchical modeling, yield coverage probabilities close to the nominal 0.95. The interval obtained from Bayesian hierarchical modeling approaches the nominal level only for 100 historical studies and remains liberal for a lower amount of historical information.

The right‐skewness of the underlying distribution increases with a decreasing binomial proportion and an increasing amount of overdispersion (panels in the lower left of each sub‐graphic). In this case, the sampled data contains many zeros and hence, the computed lower borders tend to always cover the future observation (the triangles approach 1). Hence, the prediction intervals practically aim to yield 97.5% upper prediction borders. This effect is also visible in Figure 3 and reflects a core feature of the underlying distribution, rather than being based on model misspecification.

### 5.2 Coverage Probabilities for the Simulation Setting Inspired by Long Term Carcinogenicity Studies

This part of the simulation was inspired by the estimates for the binomial proportion and overdispersion reported by Menssen and Schaarschmidt 2019 [11] for HCD obtained from long term carcinogenicity (LTC) studies run with B6C3F1 mice. 96 combinations of values for the number of historical studies H, the binomial proportion π and the amount of overdispersion ϕ were run, of which 54 combinations directly reflect the properties of the reported real life HCD (combinations of the bold faced parameter values in Table 2), whereas the remaining 42 combinations of parameter values were run in order to enhance the generalisability of the simulation.

**TABLE 2:** Parameter values used for the LTC‐inspired simulation.

Parameter	Values^a^
No. of historical studies (H)	**5**, **10**, **20**, 100
Binomial proportion (π)	**0.01**, **0.1**, **0.2**, **0.3**, **0.4**, **0.5**
Overdispersion (ϕ)^b^	**1.001**, **1.5**, **3**, 5
Historical cluster size ($n_{h}$)	**50**
Future cluster size ($n^{*}$)	**50**

*Note:* Bold numbers: Parameter values that reflect estimates obtained from real life HCD.

^a^ Simulations were run for all 96 combinations of the given parameter values.

^b^ Since the cluster sizes $n_{h}$ and $n_{*}$ are constant with $\phi \hat{=}1+\left(n_{h}-1\right)\rho \hat{=}1+\left(n^{*}-1\right)\rho$, the given values of ϕ correspond to intra‐class correlation coefficients ρ of 2.04e‐05, 0.01020, 0.04081, 0.08163.

**FIGURE 4:**
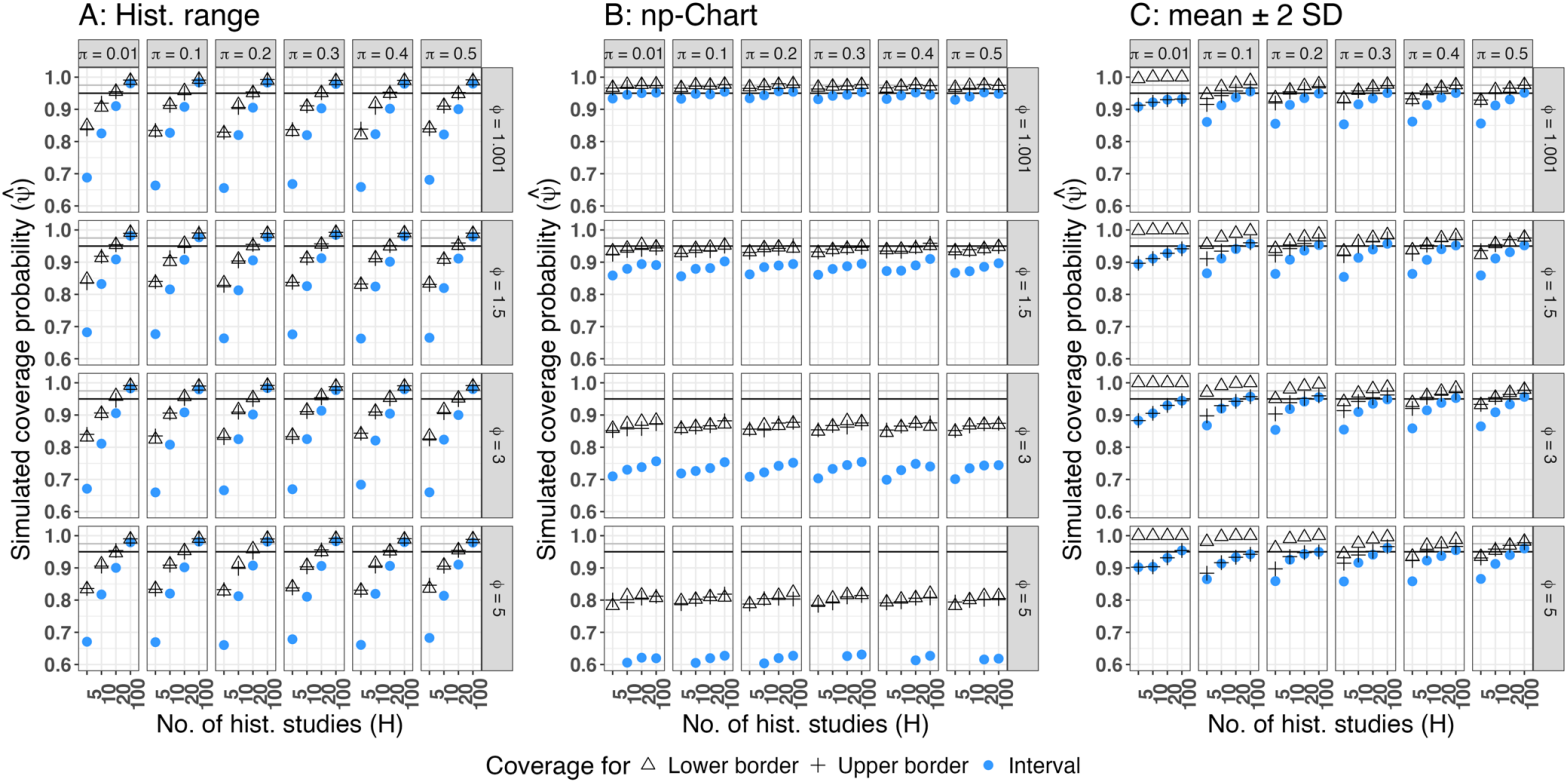
Simulated coverage probabilities of heuristical methods (LTC‐setting) (A) Historical range, (B) Np‐chart, (C) Mean ± 2 SD, *Black horizontal line*: Nominal coverage probability $\psi^{\mathrm{cp}}=0.95$, *Grey horizontal line*: Nominal coverage probability for the lower and the upper limit, if equal tail probabilities are achieved $\psi^{l}=\psi^{u}=0.975$. Coverage probabilities below 0.6 are excluded from the graphic.

Due to the relatively small cluster size, the underlying distribution is heavily discrete and can become heavily right skewed for small proportions and/or high overdispersion (see Figure 4). Hence, it is possible that in some settings (especially π=0.01) the data contains many zeros. If it happened that a sampled “historical” data set contained only zeros ($y_{h}=0\;\forall \;h=1,\ldots H$), the first observation was set to $y_{1}=0.5$ and the corresponding cluster size was set to $n_{1}=49.5$ following [11]. This procedure was applied to all frequentist methods (heuristics and prediction intervals) in order to enable the estimation process in this extreme settings, whereas otherwise, the estimates for the binomial proportion and overdispersion would become zero.

For almost all simulation settings, Bayesian hierarchical modeling faced serious convergence problems. Therefore, the simulation results are heavily unreliable and not worth reporting. In particular, the precision hyperparameter related to overdispersion is very difficult to estimate, which also depends on the prior choice. Therefore, the only Bayesian method presented in this section is the prediction interval which is calculated based on a Bayesian GLMM.

The heuristical methods (Figure 4) behaved in a similar fashion than in the MNT‐setting: With rising amount of historical control groups, the future observation is always covered (Figure 4A). In the absence of overdispersion, the np‐Chart (Figure 4B) yields coverage probabilities close to the nominal level, but with a rising amount of overdispersion, its coverage probabilities decrease below 0.6. With a rising amount of historical control groups (at least 20), the coverage probabilities of the HCL computed by the mean ± 2 SD (Figure 4C) approach the nominal level, if the underlying distribution is relatively symmetrical (low overdispersion and relatively high proportion). With increasing right‐skewness of the underlying distribution (increasing overdispersion and decreasing proportion) and/or a low amount of historical control groups (H<20) the HCL computed by the mean ± 2 SD yield coverage probabilities below the nominal level that can decrease to approximately 85%.

**FIGURE 5:**
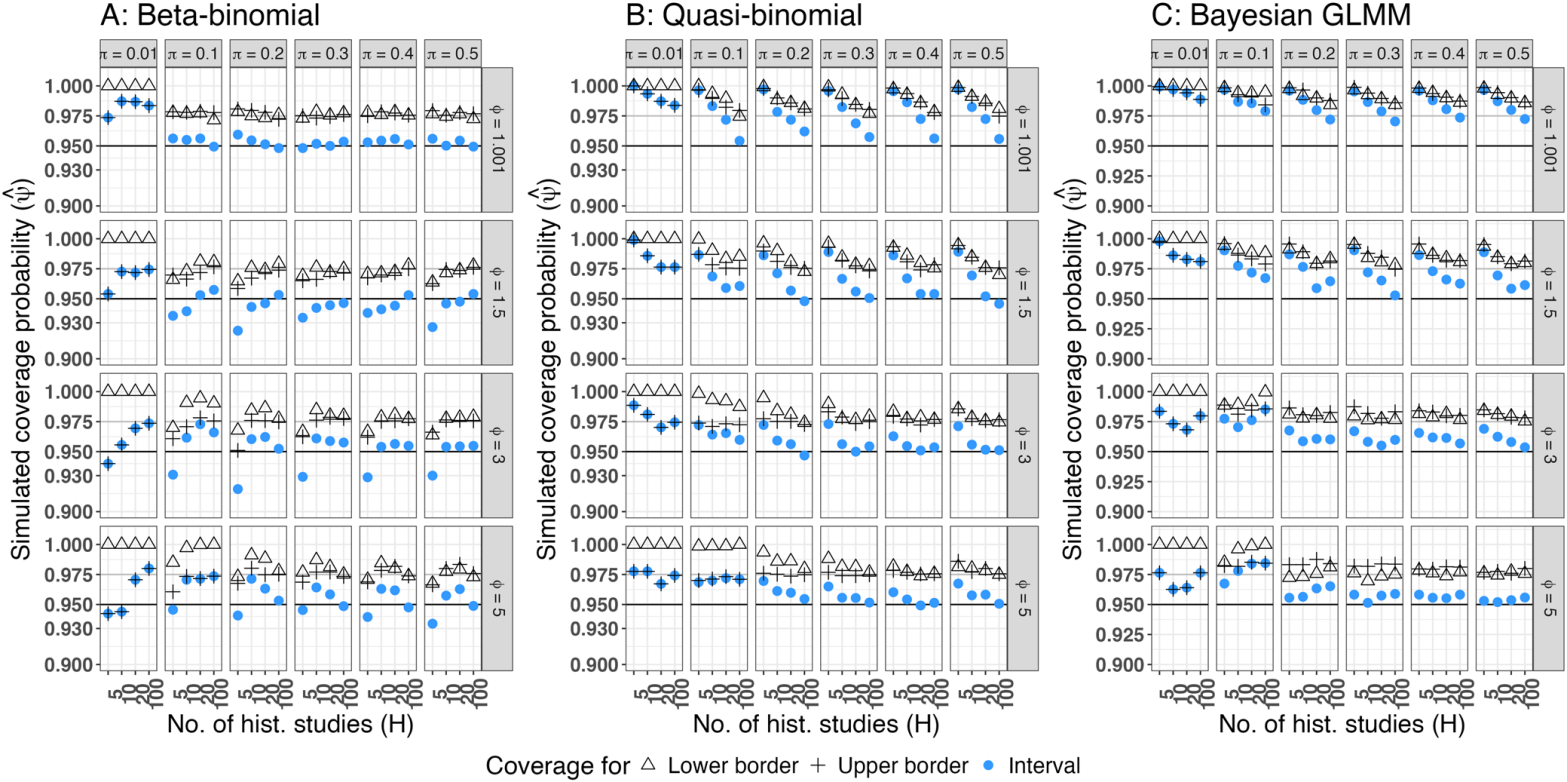
Simulated coverage probabilities of prediction intervals (LTC‐setting) (A) Calibrated beta‐binomial prediction interval, (B) Calibrated quasi‐binomial prediction interval, (C) Prediction interval obtained from a Bayesian GLMM, *Black horizontal line*: Nominal coverage probability $\psi^{\mathrm{cp}}=0.95$, *Grey horizontal line*: Nominal coverage probability for the lower and the upper limit, if equal tail probabilities are achieved $\psi^{l}=\psi^{u}=0.975$.

In the practical absence of overdispersion (ϕ=1.001), the beta‐binomial prediction interval (Figure 5A) yields coverage probabilities satisfactorily close to the nominal 0.95 (except for π=0.01), whereas the prediction intervals that are based on the quasi‐binomial assumption (Figure 5B) or drawn from a Bayesian GLMM (Figure 5C) tend to remain conservative.

For π≥0.2, moderate overdispersion ($\phi \in \left[1.5,3\right]$) and at least 10 historical control groups (H≥10) the coverage probabilities of the two frequentist prediction intervals satisfactorily approach the nominal 0.95, whereas the prediction interval obtained from the Bayesian GLMM remains slightly conservative, but may also be practically applicable in this scenario. Contrary, for high overdispersion (ϕ=5), the quasi‐binomial and the Bayesian prediction interval yield coverage probabilities satisfactorily close to the nominal level (if π≥0.2 and H≥10). In this setting, the beta‐binomial prediction interval yields coverage probabilities below the nominal level for H=5 and remains conservative for H=10,20, but approaches the nominal level for H=100.

**FIGURE 6:**
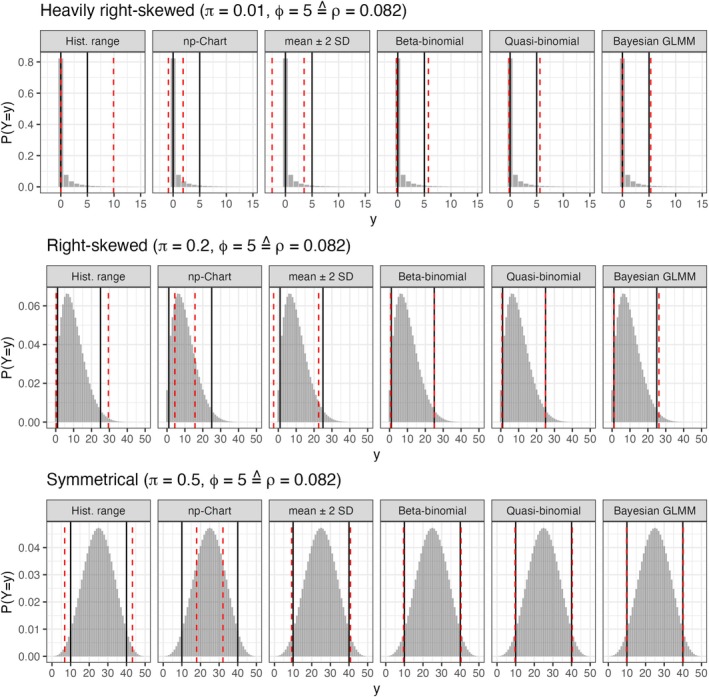
Average limits versus true quantiles of the underlying distribution (LTC‐setting). *Grey area*: Probability mass function of the underlying beta binomial distribution. *Black lines*: True underlying 2.5% and 97.5% quantiles. *Dashed red lines*: Average limits obtained from the simulation.

With increasing overdispersion ϕ and deceasing proportions π≤0.1 the right skewness of the underlying distribution increases (see Figures S1 and S2) and its lower 2.5% quantile can approach zero (see Figure 6). With other words: in this case the lower prediction border aims to approximate a true quantile that is zero or close to zero.

This is the reason why all three prediction intervals practically yield 97.5% upper prediction borders for π=0.01 and the conservativeness of the lower borders increases for π=0.1 with an increase of ϕ. This is not a misbehavior of the methodologies, but rather reflects a core feature of the underlying distribution and one should think about the application of an upper prediction border, rather than a prediction interval if many zeros are present in the data.

## 6 Application of Control Limits

The calculation of HCL based on real life data is demonstrated using HCD about the mortality of male B6C3F1‐mice in long‐term carcinogenicity studies ($n_{h}=n^{*}=50$). The data set was provided by Menssen and Schaarschmidt 2019 [11] and contains negative controls from 10 different studies run between 2003 and 2011 on behalf of the NTP. It is available via the predint R package [42] under the name mortality_HCD.

The estimate for the binomial proportion is $\hat{\pi }=0.276$ and slight overdispersion of $\hat{\phi }=1.31$ appears to be present in the data (estimated with stats::glm()). The R code for the computation of the different HCL depicted in Table 3 is available from GitHub (see link provided in Section 3.4).

**TABLE 3:** Historical control limits for the mortality of male B6C3F1‐mice obtained in negative controls of long term carcinogenicity studies at the National Toxicology Program.

Method	Lower CL	Upper CL	Interval width
Hist. range	10 (10)	21 (21)	11 (11)
np‐chart	7.47 (8)	20.12 (20)	12.65 (12)
Mean ± 2 SD	6.57 (7)	21.03 (21)	14.46 (14)
Beta‐binomial^a^	6.33 (7)	22.24 (22)	15.91 (15)
Quasi‐binomial (QP)^b^	5.77 (6)	22.71 (22)	16.94 (16)
Bayesian hierarchical^c^	7 (7)	21.025 (21)	14.025 (14)
Bayesian GLMM	6 (6)	23 (23)	17 (17)

*Note:* Numbers in brackets: Lowest and highest number of events covered by the interval.

^a^ Estimates $\hat{\pi }=0.276$ and $\hat{\rho }=0.00621$ were obtained following Lui et al. 2000 [25] (see computational details).

^b^ Estimates $\hat{\pi }=0.276$ and $\hat{\phi }=1.31$ were obtained based on stats::glm (see computational details).

^c^ A weakly informative prior $\kappa ∼\mathrm{Ga}\left(2,5\cdot 10^{-3}\right)$ was used.

The historical range yields the highest lower limit and the lowest upper limit and hence, the shortest control interval of all seven methods. Of the six methods that are aimed to cover a future observation with a probability of 0.95, the np‐chart yields the shortest interval, because it does not account for the presence of overdispersion.

Practically, the mean ± 2 SD and the prediction interval obtained from Bayesian hierarchical modeling yield the same limits [7, 21]. Both calibrated prediction intervals practically yield upper limits of 22 but the lower limit of the quasi‐binomial prediction interval is slightly lower than the beta‐binomial one (6 vs. 7). With limits [6, 23], the prediction interval drawn from a Bayesian GLMM is the widest one and hence is in line with the simulation results that revealed a slightly conservative behaviour for a comparable LTC setting of ϕ=1.5, H=10 and π=0.2 (see Figure 5C).

Note that, contrary to the description of the Bayesian hierarchical model given in Section 3.4 (which basically aims at the MNT‐inspired setting) a weakly informative prior $\kappa ∼\mathrm{Ga}\left(2,5\cdot 10^{-3}\right)$ was used for the precision hyperparameter κ. This adaption was necessary to reach convergence of the model.

## 7 Discussion

The validation of a current control group based on prediction intervals is recommended by several authors [5, 6] and since shortly, seems to be preferred by the European Food Safety Agency [10] over other (heuristical) methods. Our simulations have shown that heuristical methods do not control the statistical error to a satisfactory level and hence, cannot be recommended for the practical validation of a current control group. Contrary to this, we could show, that three of the four proposed prediction intervals satisfactorily control the statistical error, if calculated based on a model that match the statistical properties of the HCD.

### 7.1 Bayesian Prediction Intervals

The prediction intervals drawn from Bayesian hierarchical models yield coverage probabilities that mainly remain below the nominal level in the MNT‐setting (if overdispersion is present), but the model did not converge to most of the simulated data sets that mimic HCD from carcinogenicity studies (mainly if many zeros were present in the data). It is noteworthy, that in the Monte‐Carlo simulations, Bayesian hierarchical models were fit, always using the same prior $\kappa ∼\mathrm{Ga}\left(2,5\cdot 10^{-5}\right)$ regardless of the simulation setting. But, from the application to the real life example it is obvious, that this type of model needs a case to case adaption of the prior distribution to provide a reasonable domain for the hyperparameter κ (which depends on the amount of overdispersion). This might, at least partly, explain the liberality of this method. Unfortunately, the necessity for a case by case adaption of the prior distribution is far beyond the scope of most practitioners who are in charge of the reporting of HCD (mainly toxicologists that are barely trained in statistics). Hence this necessity might be a drawback for practical application.

Contrary to the Bayesian hierarchical models, the Bayesian GLMM did not have convergence problems in the LTC‐setting. But, the applied GLMM mainly yield prediction intervals with coverage probabilities above the nominal level (except for a higher amount of overdispersion). This is, because such models a priori assume between‐study variability to be present, and hence provide estimates >0 for the respective parameters, even if between‐study variability is absent in the data‐generating process.

### 7.2 Bootstrap Calibrated Prediction Intervals

The coverage probability of the calibrated intervals is mainly effected by the amount of available historical information, defined by the numbers of experimental units within each control group $n_{h}$ and number of historical studies H. In most of the cases the amount of overdispersion or the magnitude of the binomial proportion has minor impact on the coverage probability, as long as one accepts that the lower border can become zero for low proportions and/or high overdispersion such that the desired 95% prediction interval practically results in a 97.5% upper prediction limit.

For a high cluster size $n_{h}$ (as in the MNT‐setting) both calibrated prediction intervals yield coverage probabilities satisfactorily close to the nominal level, even for only five historical control groups. Contrary, for smaller cluster sizes (as in the LTC‐setting) the number of historical control groups has to be slightly higher (say 10) to enable coverage probabilities close enough to the nominal level. This difference can be explained by the fact, that with increasing numbers of experimental units within each control group ($n_{h}\to \infty$) the available information on the success rates $\pi_{h}$ rises. Consequently also their estimates ${\hat{\pi }}_{h}$ become more precise. With other words, the precision of ${\hat{\pi }}_{h}$ is practically high enough to properly estimate the magnitude of the between‐study overdispersion if $n_{h}=18000$ even for H=5. Contrary, if $n_{h}=50$ (or even lower), the information needed for the estimation of ${\hat{\pi }}_{h}$ carried by the data is heavily limited. Especially if the true (but unknown) success rate π also gets low, the data contains many zeros. This leads to two implications:

First, the estimates ${\hat{\pi }}_{h}$ become heavily imprecise. Second, the estimates for the quasi‐binomial dispersion parameter ϕ as well as for the intra‐class correlation ρ are known to be negatively biased [26, 43]. Consequently, it can happen, that the data practically appears to be underdispersed, even if it derives from a data generating process that is inherent of between‐study variation (especially, if most of the ${\hat{\pi }}_{h}$ are estimated to be zero). Since underdispersion is biologically implausible (as explained in the last paragraph of Section 2), it was necessary to restrict the estimate for the quasi binomial dispersion parameter to $\max\left(\hat{\pi },1.001\right)$. Similarly, the estimate for the beta binomial intra‐class correlation was restricted to be $\max\left(\hat{\rho },0.00001\right)$. Note that both restricted estimates have comparable magnitudes of relative bias, but the estimate for the intra‐class correlation has a higher coefficient of variation (especially if the number of available control groups H is low, see section 6 of Supporting Information S1). These higher variability might explain, that the beta‐binomial prediction interval tends to be slightly liberal for a low number of historical control groups (H=5), whereas the quasi‐binomial prediction interval behaves in a conservative fashion for this scenario.

### 7.3 Practical Handling of Overdispersed Data

The magnitude of overdispersion reflects the between‐study variation in toxicological HCD. However, systematic between‐study variation might be induced by a mixture of both, controllable causes such as different handling of experimental units by different lab personnel, as well as uncontrollable causes such as the genetic variation inherent in the population of experimental units. This means that in practice, overdispersed HCD should be handled with care in a way that the magnitude of overdispersion reflects mainly the biological variability inherent of the biological process under observation, rather than the variability that is induced by controllable causes such as different handling of experimental units by different lab personnel or the change of the supplier of the model organism [4]. However, the acceptable magnitude of between‐study overdispersion depends on the biology of the specific model organism used for a specific type of study. Consequently, a general advise on its acceptable magnitude cannot be given here, but rather has to be discussed separately for each type of study.

### 7.4 Perspectives

The bootstrap calibration procedure presented above was already applied to enable the calculation of prediction intervals drawn from linear random effects models [12] or in order to predict overdispersed count data [20]. Its application to ovderdispersed binomial data proved, that our algorithm is a general and flexible tool for the calculation of prediction intervals. Hence, for the future it is planned to exploit its potential to enable the calculation of prediction intervals for a broader range of models/distributions. Furthermore, it is possible to extend the algorithm to different types of simultaneous prediction, which will be the subject of future research.

A highly relevant application not yet covered by this manuscript is the evaluation of HCD on the base of individual (non‐aggregated) values (e.g., for the number of micronuclei for each of the six individual wells in one historical MNT‐experiment rather than for the sum of micronuclei per plate) where extra‐binomial variability is also possible within one experiment (e.g., between the wells of the same plate). For this type of data, generalized linear mixed models need to be applied. Jeske and Young [44] provided a bootstrap based procedure for the calculation of prediction intervals based on one‐way GLMMs, but to the authors knowledge, a general solution for the calculation of prediction intervals drawn from GLMMs is not yet available. In the current manuscript we showed how to derive prediction intervals from a Bayesian GLMM with one level of hierarchy. However, this approach can easily be extended to more complex designs, but these extensions will need further investigations.

## 8 Conclusions

If overdispersion is present in the data (and its magnitude is tolerable):

- Commonly applied heuristics (e.g., hist. range, np‐charts or mean ± 2 SD) do not satisfactorily control the statistical error and hence, should not be applied for the calculation of HCL.
- Symmetrical HCL do not account for possible right‐ (or left‐) skewness of the data and hence do not ensure for equal tail probabilities.
- In most of the cases, the bootstrap calibrated prediction intervals yield coverage probabilities closest to the nominal level and hence, outperform all other methods reviewed in this manuscript.
- Due to their poor statistical properties (coverage probabilities below the nominal level) and the need for a case to case adaption of prior distributions, prediction intervals drawn from Bayesian hierarchical models cannot be recommended for practical application.
- Prediction intervals computed based on Bayesian GLMM have coverage probabilities which are relatively close to the nominal level (in most cases). Because they can be easily adapted to handle more than one level of hierarchy, they are promising candidates for the application to HCD that follows a more complex design.
- With an increasing amount of overdispersion and decreasing binomial proportion, the amount of zeros in the data increases. If the amount of zeros in the data is high enough, the lower border will always cover the future observation and consequently prediction intervals (Bayesian or frequentist) practically yield 97.5% upper prediction limits. This behavior is not a drawback of the methodology, but rather reflects one of the core features of overdispersed binomial data.
- Software for the calculation of bootstrap calibrated prediction intervals is publicly available via the R package predint.

## Conflicts of Interest

The authors declare no conflicts of interest.

## Supporting information


**Data S1:** Supporting Information.

## Data Availability

The data that support the findings of this study are available on request from the corresponding author. The data are not publicly available due to privacy or ethical restrictions.
