# Supplementary material for: Prediction Intervals for Overdispersed Binomial Endpoints and Their Application to Toxicological Historical Control Data
Source: Pharm Stat. 2025 Sep 14;24(5):e70033. doi: 10.1002/pst.70033 (PMC12433933; doi:10.1002/pst.70033)
Supplement: Supplementary file 1 — Data S1: Supporting Information. [file PST-24-0-s001.pdf]

Supplementary materials: Prediction intervals for overdispersed binomial endpoints and their application to toxicological historical control data

Max Menssen[1*], Jonathan Rathjens[2],

*: Corresponding author

1: Department of Biostatistics, Leibniz University Hannover

2: Early Development Statistics, Chrestos Concept GmbH & Co. KG, Essen

1 Shape of sampling distributions

The data shown in figures 1 and 2 was generated with `predint::rqbinom()`. Each histogram is based on 10000 data points. Note that with an increase of overdispersion ϕ and/or a decrease of the binomial proportion π, the right skeweness of the underlying distribution increases.

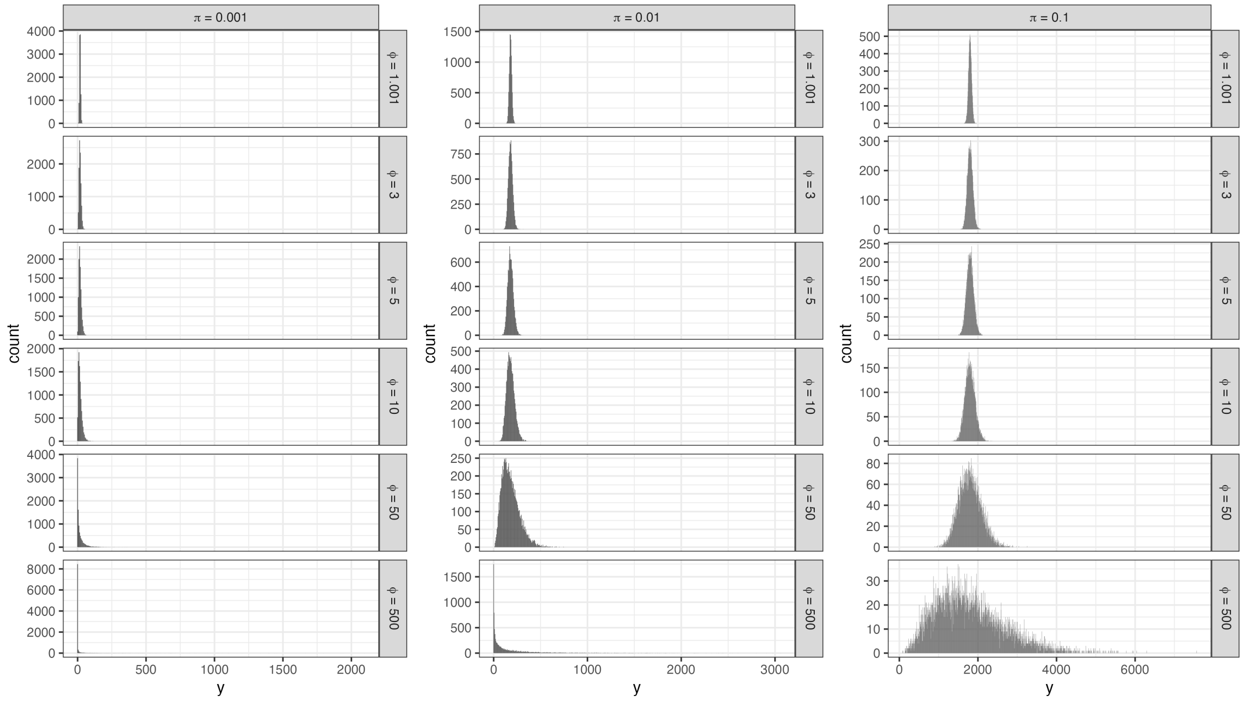

Figure 1: Histograms for the empirical distributions of overdispersed binomial data used in the MNT-setting of the Monte-Carlo simulation ($n_h = n^* = 18000$).

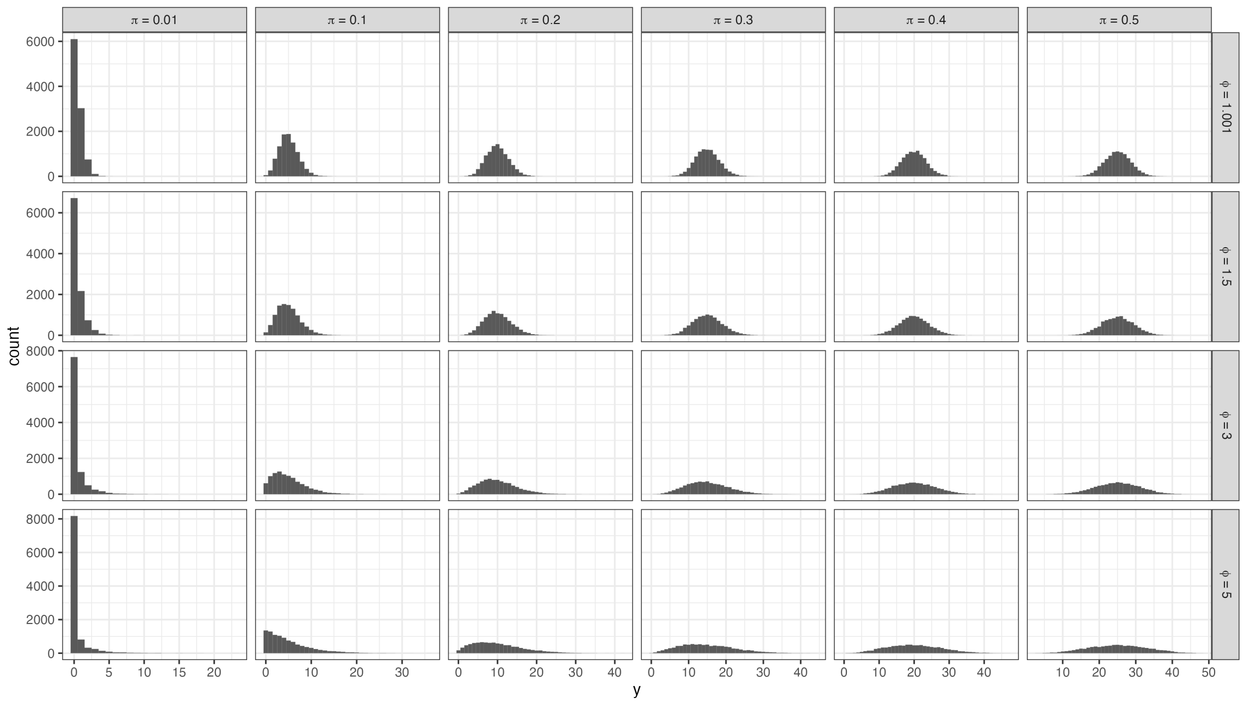

Figure 2: Histograms for the empirical distributions of overdispersed binomial data used in the LTC-setting of the Monte-Carlo simulation ($n_h = n^* = 50$)

2 Mean ± 2 SD: Coverage probabilities for different cluster size

The coverage probabilities for the application of the mean ± 2 SD to data with different cluster sizes were simulated for the LTC setting. The only difference to the simulation in the main manuscript was, that the historical and current cluster sizes n_{hs} and n^*_s were sampled from a discrete uniform distribution with min=10 and max=100 (with $s = 1, \dots, 5000$ as the index for each simulation step per parameter combination).

Simulations were run for two different types of application: The calculation of the limits of response scale, ignoring that the cluster size was different (figure 3A) and on proportion scale (figure 3B). For the latter, the number of observations was divided by the cluster size $\hat{\pi}_{hs} = y_{hs}/n_{hs}$ and the mean and SD were also calculated on proportion scale $\bar{\hat{\pi}}_s = \sum_h \hat{\pi}_{hs}/H_s$ and $sd(\hat{\pi})_s = \sqrt{\frac{(\bar{\hat{\pi}}_s - \hat{\pi}_{hs})^2}{H_s - 1}}$.

Due to the different cluster size, the interval calculated on response scale lacks a formal definition of the statistical error and hence, obviously does not yield coverage probabilities that systematically approach the nominal 0.95 with an increase of historical studies.
The interval on proportion scale, that seems to account for the problem of different cluster sizes, does not systematically approach the nominal 0.95, too. This can be explained by the fact, that the uncertainty of the estimate for the proportion depends also on the cluster size, such that

$$\widehat{var}(\hat{\pi}_h) = \widehat{var}(y_h/n_h) = \widehat{var}(y_h)\frac{1}{n_h^2} = \hat{\phi} n_h \hat{\pi}_h (1-\hat{\pi}_h)\frac{1}{n_h^2} = \frac{\hat{\phi}\hat{\pi}_h(1-\hat{\pi}_h)}{n_h}$$

but the application of the mean $\pm$ 2 SD explicitly depends on the assumption of variance homogeneity of the estimates for the binomial proportion ($\hat{\pi}_h$ and $\hat{\pi}^*$).
Note, that the two bootstrap calibrated (frequentist) prediction intervals shown in the main manuscript explicitly account for different cluster sizes, and hence are also be applicable in this scenario.

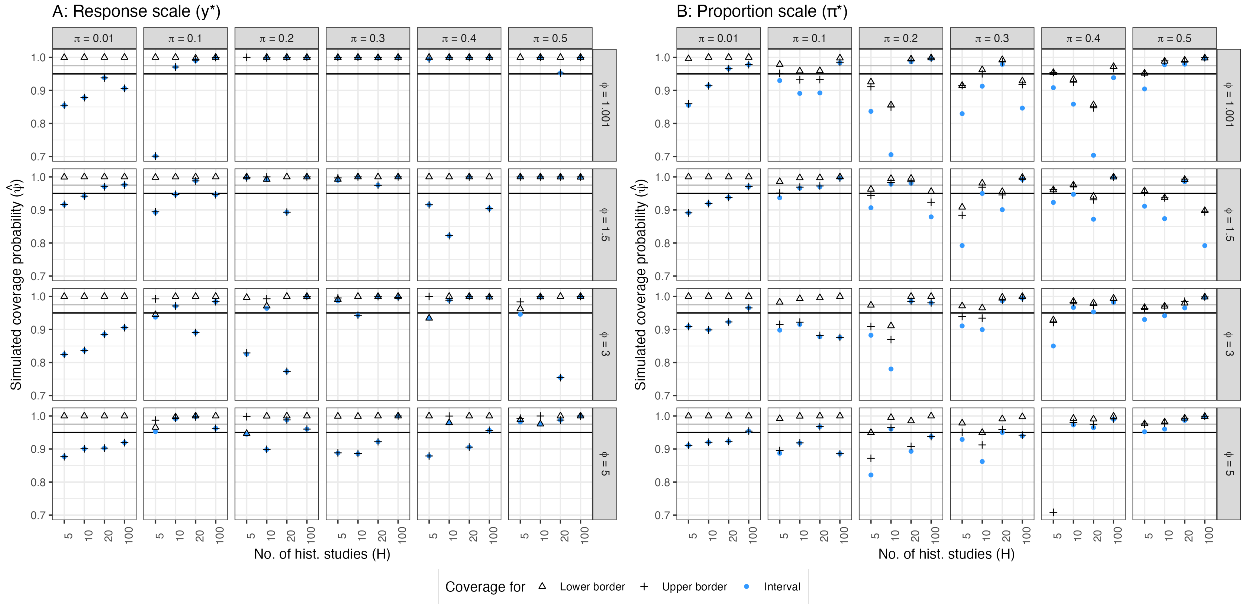

Figure 3: Coverage probabilities for the mean $\pm$ 2 SD for different cluster sizes. The historical and current cluster sizes n_h and n^* were randomly sampled from a discrete uniform distribution with min=10 and max=100

3 Sampling of overdispersed binomial data

3.1 Beta-binomial

For beta-binomial data with $i = 1, \dots, I$ clusters, the variance is

$$var(y_i) = n_i \pi (1 - \pi)[1 + (n_i - 1)\rho]$$

with ρ as the intra-class correlation coefficient

$$\rho = \frac{1}{1 + a + b}.$$

For given values of n_i, π and ρ, beta-binomial data can be samples as follows: Define $(a + b)$ as

$$(a + b) = \frac{1 - \rho}{\rho}$$

where $a = \pi(a + b)$ and $b = (a + b) - a$. Then, the binomial proportion for each cluster is sampled from the beta distribution

$$\pi_i \sim beta(a, b)$$

and the number of successes for each cluster is sampled to be

$$y_i \sim Bin(n_i, \pi_i).$$

In this parametrization, $E(\pi_i) = \pi = a/(a + b)$ and $E(y_i) = n_i \pi$. This sampling algorithm is implemented in `predint::rbbinom()`.

3.2 Quasi-binomial

Under the quasi-binomial assumption, the overdispersion is constant between the clusters, such that

$$var(y_i) = n_i \pi (1 - \pi)\phi.$$

For given values of n_i, π and ϕ data that exhibits constant overdispersion can be sampled as follows: Define

$$a_i + b_i = \frac{\phi - n_i}{1 - \phi}$$

where $a_i = \pi(a_i + b_i)$ and $b_i = (a_i + b_i) = a_i$. Then, the binomial proportion for each cluster is sampled from the beta distribution

$$\pi_i \sim beta(a_i, b_i)$$

and the number of successes for each cluster is sampled to be

$$y_i \sim Bin(n_i, \pi_i).$$

In this parametrization, $E(\pi_i) = \pi$ and $E(y_i) = n_i\pi$. This sampling algorithm is implemented in `predint::rqbinom()`.

3.3 Constant cluster size

In the case of constant cluster size all n_i are equal and hence the quasi-binomial and the beta-binomial assumptions are not in contradiction to each other. In this special case, one can relate the quasi-binomial ϕ and the beta-binomial intra-class correlation by

$$\rho \widehat{=} \frac{\phi - 1}{n - 1}$$

to each other.

Therefore, the following R code

```
> set.seed(123)
> predint::rqbinom(n=5, size=50, prob=0.1, phi=3)


> set.seed(123)
> predint::rbbinom(n=5, size=50, prob=0.1, rho=2/49)
```

yields exactly the same data set

```
  succ fail
1    6   44
2    4   46
3   18   32
4    6   44
5    3   47
```

4 Overdispersion in real life HCD

In 1982 Tarone published HCD from 70 experiments about the number of female F344 rats within each control group that developed endometrial stromal polyps. If one fits a generalized linear model (based on the quasi-binomial assumption) to this data set, the estimate for the dispersion parameter is $\hat{\phi} = 2.06$. Carlus et al. 2013 provide four HC data sets on the survival incidence of Wistar Han rats in long-term carcinogenicity studies out of which two showed clear signs of overdispersion ($\hat{\phi} = 1.35$ and $\hat{\phi} = 1.68$).

Also for count data HCD that exhibit overdispersion was published by several authors. Menssen et al. 2024 analyzed 49 real life HC data sets from the Ames test, of which 40 were indeed overdispersed. Also Levy et al. 2019 reported HCD from the Ames test of which, according to Menssen et al. 2024, 12 out of 18 data sets show signs of overdispersion. Tarone 1982 published HCD from 66 microbial mutagenesis assays, which according to Menssen et al. 2024 is also overdispersed ($\hat{\phi} = 3.18$).

The presence of overdispersion reflects the amount of between-study variability which can be found also in assays with continuous endpoints. Tug et al. 2024 reported HCD from the comet assay which exhibits substantial between study variability. This findings are in line with the HCD from four different assays (Pig-a gene mutation assay, erythrocyte-based micronucleus test, transgenic rodent gene mutation assay, in vivo alkaline comet assay) reproted by Dertinger et al 2023.

5 Properties of the asymptotic prediction interval of Menssen and Schaarschmidt 2019

Figure 4 depicts the ability to cover the central 95 % of the underlying distribution of the bootstrap calibrated asymptotic prediction interval for the quasibinomial assumption of Menssen and Schaarschmidt 2019 (Nelsonphi1_bisec). The calibration of this interval follows the "classical" alpha-calibration approach and hence calibrates both interval borders together (and not individually). Consequently, this interval yields prediction borders that are systematically below the true quantiles of the underlying distribution (if this distribution

is right-skewed) and hence, does not account for equal tail probabilities.

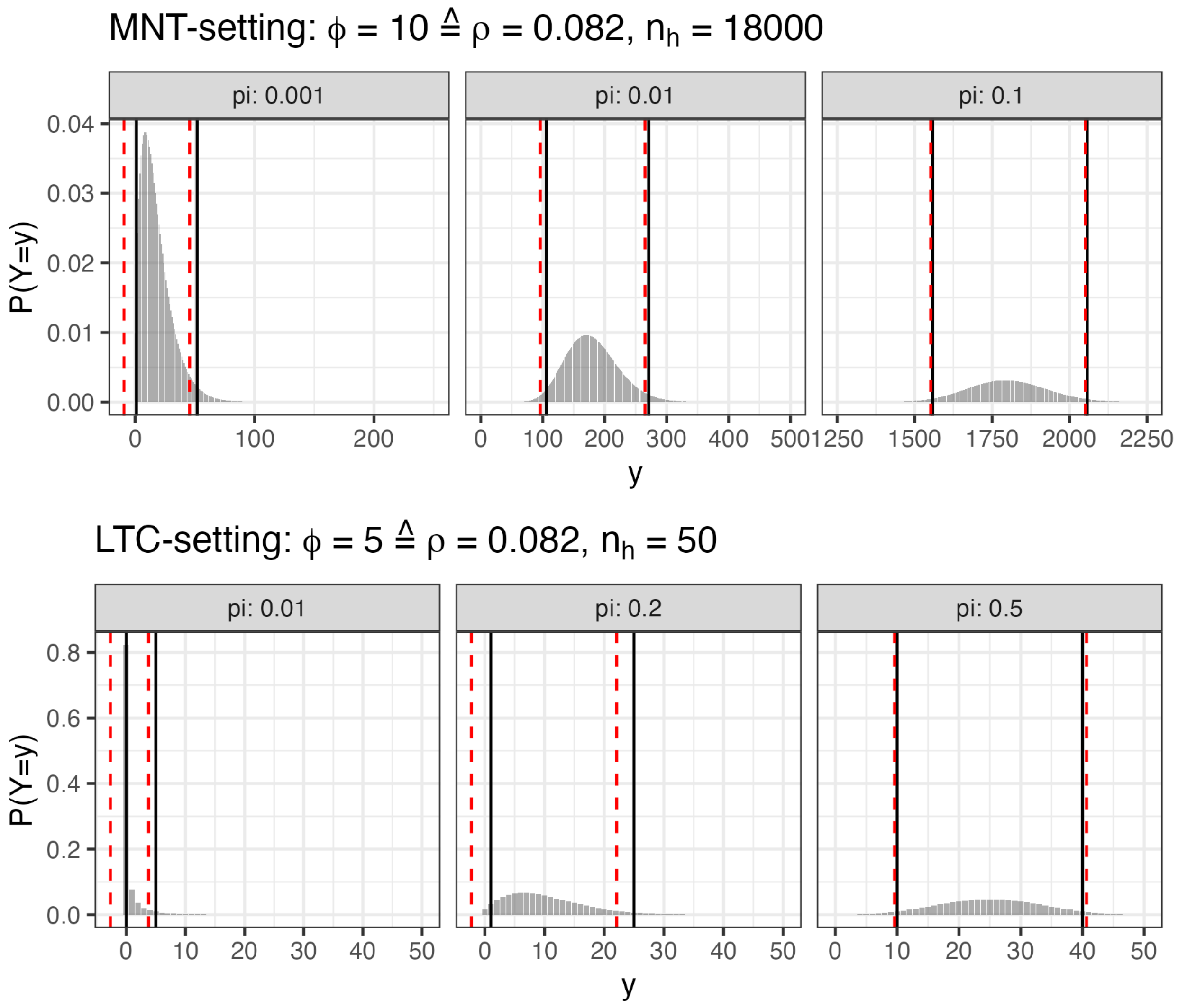

Figure 4: Average limits vs. true quantiles of the underlying distribution for the bootstrap calibrated prediction interval of Menssen and Schaarschmidt 2019. Grey area: Probability mass function of the underlying beta binomial distribution. Black lines: True underlying 2.5 % and 97.5% quantiles. Dashed red lines: Average limits for $H = 100$ obtained from the simulation.

6 Properties of the estimates for intra-class correlation and dispersion parameter

The assessment of the properties of the restricted estimates for intra-class correlation and dispersion parameter was carried out based on the LTC-setting. For each combination of the parameters (given in tab. 2 of the main manuscript), $S = 1000$ data sets were drawn. From each of these data sets $\hat{\rho}_s^r = max(0.00001, \hat{\rho}_s)$ and $\hat{\phi}_s^r = max(1.001, \hat{\phi}_s)$ were estimated. For each of the restricted estimates the relative bias (red triangles in fig. 5) was calculated as

$$RB^{\hat{\phi}^r} = \frac{\sum_s \frac{\hat{\phi}_s^r}{\phi}}{S}$$

and

$$RB^{\hat{\rho}^r} = \frac{\sum_s \frac{\hat{\rho}_s^r}{\rho}}{S}.$$

The coefficient of correlation (see fig. 6) for both of the restricted estimates was calculated to be

$$CV^{\hat{\phi}^r} = \frac{\sqrt{\frac{1}{S}\sum_s(\hat{\phi}_s^r - \bar{\hat{\phi}}^r)^2}}{\bar{\hat{\phi}}^r}$$

and

$$CV^{\hat{\rho}^r} = \frac{\sqrt{\frac{1}{S}\sum_s(\hat{\rho}_s^r - \bar{\hat{\rho}}^r)^2}}{\bar{\hat{\rho}}^r}$$

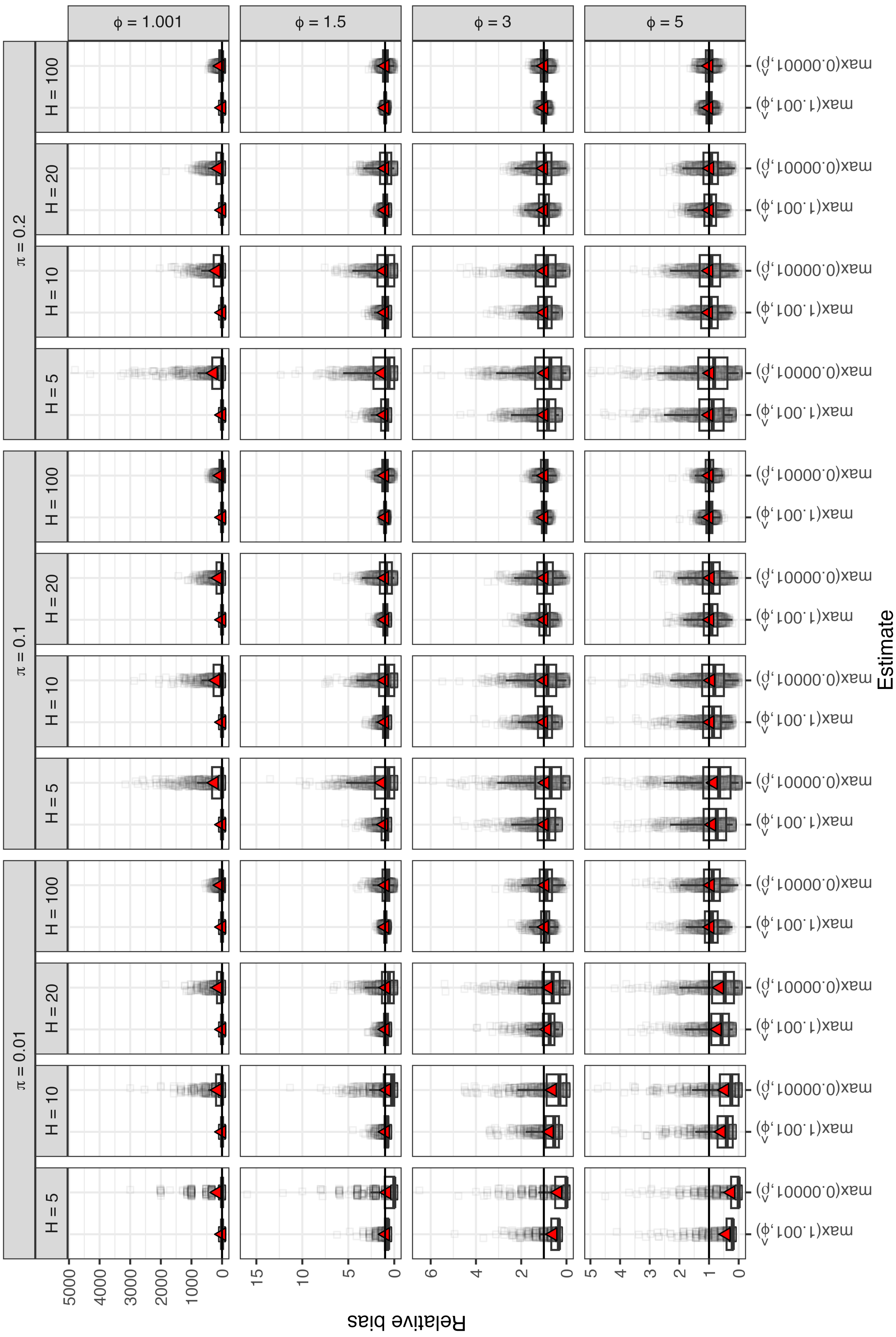

Figure 5: Relative bias of the restricted estimates for the intra-class correlation and the dispersion parameter. Grey dots: Ratio between estimate and true parameter $max(1.001, \hat{\phi}_s)/\phi$ or $max(0.00001, \hat{\phi}_s)/\phi$. Red triangles: Average ratios (relative bias).

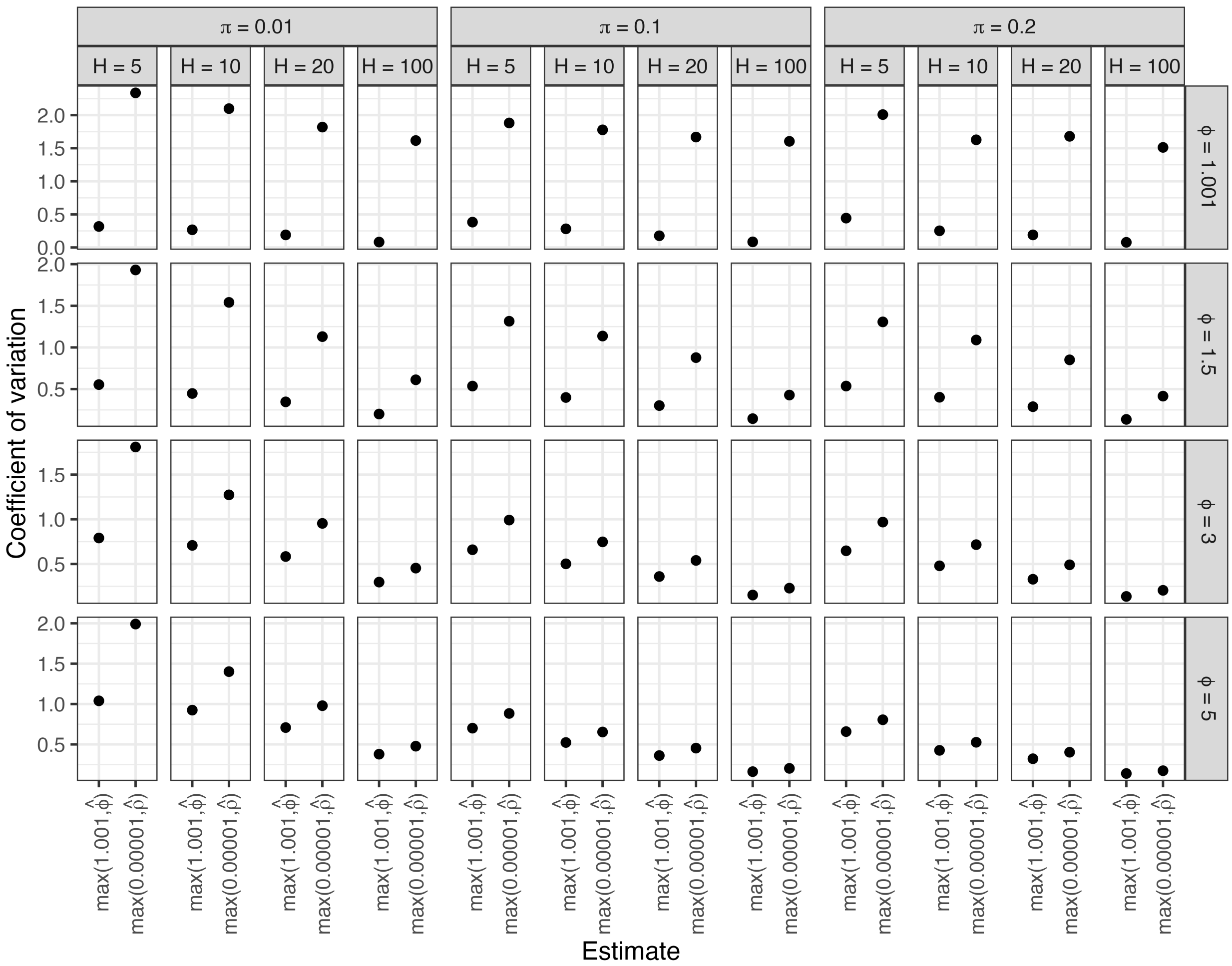

Figure 6: Coefficient of variation for the restricted estimates for the intra-class correlation and the dispersion parameter.

7 References

Carlus, M., Elies, L., Fouque, M. C., Maliver, P., Schorsch, F. (2013). Historical control data of neoplastic lesions in the Wistar Hannover Rat among eight 2-year carcinogenicity studies. Experimental and Toxicologic Pathology, 65(3), 243-253.

Dertinger, S. D., Li, D., Beevers, C., Douglas, G. R., Heflich, R. H., Lovell, D. P., Roberts D. J., Smith R., Uno Y., Williams A., Witt K. L., Zeeler A., Zhou, C. (2023). Assessing the quality and making appropriate use of historical negative control data: A report of the

International Workshop on Genotoxicity Testing (IWGT). Environmental and Molecular Mutagenesis, 1-22.

Levy, D. D., Zeiger, E., Escobar, P. A., Hakura, A., Bas-Jan, M., Kato, M., Moore M. M., Sugiyama, K. I. (2019). Recommended criteria for the evaluation of bacterial mutagenicity data (Ames test). Mutation Research/Genetic Toxicology and Environmental Mutagenesis, 848, 403074.

Menssen, M., Dammann, M., Fneish., F., Ellenberger., D., Schaarschmidt, F., (2024): Prediction intervals for overdispersed Poisson data and their application in medical and pre-clinical quality control. arXiv:2404.05282 (under review in Pharmaceutical Statistics).

Tarone, R. E. (1982a). The use of historical control information in testing for a trend in proportions. Biometrics, 215-220.

Tarone, R. E. (1982b). The use of historical control information in testing for a trend in Poisson means. Biometrics, 457-462.

Tug, T., Duda, J. C., Menssen, M., Bruce, S. W., Bringezu, F., Dammann, M., Frötschl, R., Harm, V., Ickstadt, K., Igl, B-W., Jarzombek, M., Kellner, R., Lott, J., Pfuhler, S., Plappert-Helbig, U., Rahnenführer, J., Schulz, M., Vaas, L., Vasquez., M., Ziegler., V., Ziemann, C. (2024). In vivo alkaline comet assay: Statistical considerations on historical negative and positive control data. Regulatory Toxicology and Pharmacology, 148, 105583.
